# AgroXAI: a multi-XAI deep learning system for explainable multi-species plant disease classification

**DOI:** 10.3389/fpls.2026.1965848

**Published:** 2026-10-14

**Authors:** Sathish Kumar K., G. N. Balaji

**Affiliations:** School of Computer Science and Engineering, Vellore Institute of Technology, Vellore, India

**Keywords:** deep learning, explainability validation, explainable artificial intelligence (XAI), multi-XAI fusion, plant leaf disease detection, Swin transformer

## Abstract

Automated plant disease classification can benefit from models that provide both predictive performance and interpretable evidence for their decisions. This study presents AgroXAI, a Multi-XAI deep learning framework for explainable multi-species plant disease classification under a controlled, leakage-aware evaluation protocol. A publicly available dataset containing 2,815 leaf images from seven plant species was organized into 14 joint species–health classes and partitioned into fixed group-disjoint training, validation, and held-out test sets containing 1,970, 422, and 423 images, respectively. Preprocessing and architecture selection were conducted using only the training and validation partitions, leading to the AgroX-Swin classifier based on Swin Transformer-Tiny with 384 × 384 Original RGB inputs. Explainability was quantitatively evaluated using Grad-CAM++, SHAP, LIME, and their complementary fusions on a predefined class-balanced subset of 42 held-out images. Across five independently initialized training runs, AgroX-Swin achieved a mean held-out accuracy of 87.19% and macro-F1 of 0.8300 ± 0.0122, while the five-model probability ensemble achieved 87.94% accuracy and 0.8355 macro-F1. Deterministic aggregation of the same 14-class predictions yielded 99.29% species-recognition accuracy and 88.42% binary health-status accuracy, with a diseased-class F1-score of 0.9160. Temperature scaling reduced the representative model's expected calibration error from 0.0792 to 0.0310. At the 20% perturbation level, Grad-CAM++ + LIME produced the largest mean confidence drop (0.2540), while Grad-CAM++ showed the highest transformation stability (0.8371) and SHAP + LIME showed the highest attribution-burden stability (0.7954), demonstrating complementary rather than uniformly superior behavior among individual and fused explanation methods. Model-randomization analysis further examined the dependence of Grad-CAM++ explanations on learned model parameters. AgroXAI provides an integrated framework combining leakage-aware model development, multi-seed training-stability assessment, held-out classification, calibration, statistical uncertainty analysis, and quantitative evaluation of complementary XAI methods. The resulting attribution maps are interpreted as prediction-relevant visual explanations rather than validated lesion localization or quantitative disease-severity estimates. External robustness and real-world agricultural generalization remain to be established using independently acquired field datasets.

## 1 Introduction

### 1.1 Background and motivation

Food security, socioeconomic development, and sustainable resource use are vital aspects of agriculture worldwide. Despite available registered products, plant diseases still pose a significant threat to agricultural production, leading to losses in yield and product quality. Traditional methods for assessing plant diseases mainly rely on visual inspection and expert diagnosis in the field, which are laborious, time-consuming, subjective, and challenging, especially for large-scale crops. For this reason, image-based automated plant-disease recognition is becoming more popular and is a key element of intelligent and precision agriculture (Sajitha et al., 2024; Shafik et al., 2024; Shoaib et al., 2023; Sunil et al., 2023; Upadhyay et al., 2025; Xu et al., 2024; Zhao J, et al., 2025).

Recent developments in Deep Learning (DL) and Computer Vision (CV) now make automated recognition of plant diseases from leaf images possible. Deep neural networks learn discriminative representations related to color variation, texture, lesion appearance, lesion morphology, and structural patterns, rather than relying solely on handcrafted features. Another feature that can facilitate model development when agricultural data is relatively small is transfer learning and pre-trained visual backbones. Therefore, there is growing interest in novel deep learning architectures, such as convolutional architectures, attention mechanisms, and others, for automated plant-disease classification (Alirezazadeh et al., 2023; Ashurov et al., 2025; Atila et al., 2021; Hang et al., 2024; Shafik et al., 2024).

Despite these advances, plant disease classification still depends on image characteristics and experimental design. The visual representations learnt by a model can be affected by different illumination, orientation, scale, background composition, leaf morphology, and symptom appearance. Patterns may be similar under different disease conditions, and samples from different Disease and Disease-free varieties of the same species may have similar morphological characteristics. Another methodological issue is including multiple source images in the model development and evaluation partitions that are exact copies of each other, or very similar, which can cause information leakage and optimistically biased estimates of held-out predictive performance. Hence, sanitizing the data, partitioning with leakage in mind, carefully choosing a model and setting the final held-out test set is also crucial to an evaluation protocol (Sajitha et al., 2024; Sunil et al., 2023; Xu et al., 2024; Zhao J, et al., 2025).

Another methodological factor that can affect classification accuracy is image preprocessing. Removing the background and leaves can reduce noise and background information irrelevant to the task, and deep image segmentation models can help extract the foreground (Qin et al., 2020). However, foreground isolation may be too aggressive and remove contextual, boundary, color, and/or structural information that may assist classification. Hence, the effectiveness of segmentation-based preprocessing in enhancing downstream classification is not known *a priori* and should be tested empirically. Likewise, it is not always possible to predict which deep-learning model will be most effective across the full range of agricultural datasets. Therefore, comparative pre-processing and architecture screening based on a predetermined validation protocol are crucial before final model evaluation.

Predictive performance is not the only indicator of how a deep-learning model reaches an individual classification. Explainable AI (XAI) techniques can generate model-specific data on image regions or features that contribute to a prediction. Grad-CAM and Grad-CAM++ offer gradient-based explanations of the spatial distribution of predictions; SHAP offers additive explanations of the features that contribute to predictions; and LIME approximates local prediction behavior with interpretable surrogate models (Chattopadhay et al., 2018; Lundberg et al., 2020; Ribeiro et al., 2016; Selvaraju et al., 2020). More and more studies on plant disease recognition use XAI techniques to enhance the transparency and interpretability of model predictions (Kaler and Kaur, 2025; Nahiduzzaman et al., 2023; Natarajan et al., 2024; Nazri and Razak, 2026; Rashid et al., 2025).

Each XAI method, however, relies on different mathematical techniques and may produce different attribution patterns for the same image. This means one explanation method can only provide method-specific insight into model behavior, while complementary methods can offer different perspectives on the visual evidence influencing the prediction. However, producing a visually plausible map is not enough to be considered an explanation; it must also be stable, faithful, and biologically valid. To characterize attribution, quantitatively evaluate its relevance to model predictions, its consistency under controlled transformations, and its dependence on learned model parameters (Kaler and Kaur, 2025; Nazri and Razak, 2026; Özüpak et al., 2025; Verma et al., 2025a).

Importantly, it is necessary to distinguish model attribution from biological lesion localization and disease-severity estimation. With suitable lesion annotations and ground-truth data, segmentation-based methods can quantitatively estimate lesion area or disease severity (Polly and Devi, 2024; Singh et al., 2025; Yang et al., 2024; Zhao W, et al., 2025). In contrast, XAI attribution maps highlight regions of an image that contribute to a model’s prediction but are not necessarily interpretable as disease lesions. Highly attributed regions may be lesion-like regions, Disease-free leaf morphology, venation, boundaries, texture, color, etc., that are discriminative. However, because no independent experts annotated the lesion masks, the present study treats the XAI maps as visual explanations relevant to the prediction task and the spatial summaries as an approximation of the relative burden of visual explanations, not as validated lesion boundaries or quantitative estimates of biological disease severity.

Motivated by these considerations, this study proposes a new evaluation framework, AgroXAI, which is not a new backbone or a new XAI algorithm. The main learning task consists of 14 joint species–health classes, each composed of 2 classes: one with a particular species and one with a particular health status (Disease or Disease-free). The experimental workflow includes dataset sanitization and leakage auditing; partitioning into training, validation, and test groups; comparing original RGB preprocessing with U²Net preprocessing; screening architectures and selecting models; and running the training process 5 times independently. The experimental workflow includes dataset sanitization, leakage audit, group-disjoint training, validation, and test partitioning; a controlled comparison of original RGB preprocessing with U²Net preprocessing; architecture screening and model selection; and five independent training runs, each on a held-out independent test dataset. The resulting AgroX-Swin uses a Swin Transformer-Tiny backbone with a 384 × 384 input. These mappings are in addition to the 14-class predictions and are not hierarchical or multi-stage classifiers. Still, they are deterministically mapped to seven species categories and two health-status categories for multi-level evaluation.

Complementary Grad-CAM++, SHAP, and LIME analyses are used to examine prediction-relevant visual attribution, together with individual, pairwise, and three-way Multi-XAI fusion analyses. Beyond qualitative visualization, the explanation analysis considers perturbation fidelity, transformation-based stability, model-randomization behavior, fusion sensitivity, cross-explainer spatial agreement, attribution-burden stability, and computational cost. AgroXAI therefore integrates leakage-aware model development, multi-seed training-stability assessment, held-out classification, calibration, statistical uncertainty analysis, and quantitative evaluation of complementary XAI methods. Because evaluation is confined to a held-out subset of the same public dataset, cross-dataset performance and real-world field generalization are not claimed and remain to be established using independently acquired external datasets.

### 1.2 Organization of the paper

The rest of this paper is organized as follows: Section 2 provides an overview of related research in the field of deep learning-based plant disease classification, attention- and transformer-based plant disease recognition, Explainable Artificial Intelligence (XAI), Multi-XAI approaches, and segmentation-based plant disease assessment, and highlights the methodological gaps that are tackled by the proposed framework. Section 3 introduces the AgroXAI approach, which involves organizing the datasets and ensuring their integrity, leakage-aware group-disjoint partitioning, the ablation of preprocessing, architecture screening and model selection, the assessment of stability of the datasets during multiple training, held-out evaluation, predictive calibration, statistical uncertainty analysis, and complementary explanation and fusion procedures of Grad-CAM, SHAP, and LIME. It also outlines quantitative XAI assessment with perturbation fidelity, transformation-based stability, model-randomization sanity testing, cross-explainer agreement, fusion sensitivity, attribution-burden analysis, and computational assessment. Section 4 introduces and discusses experimental results on preprocessing and architecture selection, multi-seed training stability, multi-level classification and error analysis, predictive calibration and statistical uncertainty, quantitative XAI fidelity and stability, model randomization analysis, cross-explainer agreement, fusion sensitivity, attribution-burden analysis, and computational trade-offs. Section 5 summarizes the main study limitations: the study lacks independent external validation and expert-annotated lesion ground truth to validate attribution maps biologically. Finally, Section 6 concludes the chapter with a summary of the key findings and discusses directions for future research, such as conducting external field testing and having teachers and developers review explanation outputs.

## 2 Related works

### 2.1 Deep learning-based plant disease classification

Automated plant disease recognition (APDR) is a crucial application of Deep Learning (DL) in agricultural image analysis. Unlike traditional machine-learning methods that rely heavily on manually engineered features, deep neural networks can extract discriminative features directly from leaf images, such as color variations, texture, lesion morphology, and structural abnormalities. The field of plant disease recognition now uses convolutional neural networks (CNNs), transfer learning with other CNN models, and other deep architectures across various crops and diseases (Sajitha et al., 2024; Shafik et al., 2024; Shoaib et al., 2023; Sunil et al., 2023; Upadhyay et al., 2025; Zhao et al., 2024).

Transfer learning further aids plant-disease recognition by using pre-trained visual representations rather than learning all image representations from available agricultural data alone. EfficientNet-based models, Residual architectures, and Lightweight CNNs have thus far been explored to strike an appropriate balance between prediction ability and compute cost (Ashurov et al., 2025; Atila et al., 2021; Hang et al., 2024). However, dataset composition, image quality, background characteristics, class imbalance, and acquisition conditions can affect classification accuracy. Thus, evaluating plant-disease classifiers should account for dataset and experimental design characteristics, as well as overall classification accuracy (Xu et al., 2024).

Another factor that can affect downstream classification is pre-processing. In the classification process, one of the most common methods for filtering out background information is foreground extraction and leaf segmentation (Qin et al., 2020). Researchers have applied salient-object detection and foreground-extraction methods based on nested U-structures to U²Net, and have explored segmentation methods like U-Net for plant-disease analysis (Kotwal et al., 2024; Singh et al., 2025). The problem with segmentation-based pre-processing is that if the segmentation process changes or loses class-relevant information, it doesn’t necessarily help the post-processing classification. This encourages empirical testing and comparison of different preprocessing alternatives under a uniform experimental protocol, rather than assuming better performance from “ foreground” segmentation in general.

### 2.2 Attention- and transformer-based plant disease recognition

Recent studies of attention mechanisms aimed at highlighting meaningful visual aspects in representation learning have begun to be explored in plant-disease recognition. In channel attention, informative feature channels are emphasized; in spatial attention, the model focuses on the relevant spatial region. These mechanisms have been studied to better model disease-related attributes that are subtle or diffuse in space (Alirezazadeh et al., 2023; Hang et al., 2024; Mythili et al., 2026).

When a disease’s association with visual patterns is localized or heterogeneous across the leaf, attention-enhanced CNNs and related architectures can be highly beneficial. Some previous works combine attention mechanisms with feature extraction, segmentation, and classification, showing the feasibility of applying attention-based learning to agricultural image analysis (Alirezazadeh et al., 2023; Kotwal et al., 2024). However, effectiveness depends on the dataset, task, and experimental setup. This means one should not assume that a specific attention mechanism will yield the best results when choosing an architecture, but should instead proceed with a modeling approach based on the architecture and a known validation protocol.

In addition to local (conv.) representation learning, attention-based visual modeling also enables learning relationships between spatial regions in images that contribute to the representation. This property is important for joint classification of multi-species plant diseases, which requires a classifier to assess visual properties according to plant species and health status. Therefore, alternative architectures should be tested in the same experimental setup, not because they necessarily perform better on held-out data, but because they might.

### 2.3 Explainable artificial intelligence for plant disease analysis

While deep learning’s ability to predict performance in plant-disease classification is improving, the black-box nature of deep-learning models remains important to consider for plant image analysis in agriculture. There is no guarantee that, if a classification is correct, then a model assumes that the plant has a specific set of characteristics that are important to its prediction, or that it is based on characteristics that are potentially irrelevant to the prediction. In plant health, Explainable Artificial Intelligence (XAI) methods are increasingly used to analyze the evidence behind an individual prediction (Jafar et al., 2024; Kaler and Kaur, 2025; Nahiduzzaman et al., 2023; Natarajan et al., 2024; Upadhyay et al., 2025). In contrast, dedicated segmentation architectures can explicitly delineate disease-affected regions in plant leaf images (Zhao W, et al., 2025).

Gradient-based attribution is one method for exploring spatial evidence in image classification. Grad-CAM and Grad-CAM++ share the same idea and aim to identify influential spatial regions, except that the latter uses weighted positive partial derivatives to produce class-discriminative spatial attribution (Chattopadhay et al., 2018; Selvaraju et al., 2020). These approaches provide visual representations of image regions that help a classifier make a prediction. Still, the resulting attribution maps do not directly annotate biological lesions; instead, they provide model-centered explanations.

Model-agnostic approaches offer complementary views of prediction behavior. LIME simulates an instance’s predictability by learning a simple model, while model SHAP uses additive principles of feature attribution to characterize contributions to predictions (Lundberg et al., 2020; Ribeiro et al., 2016). Model-agnostic approaches offer complementary views of prediction behavior. LIME simulates an instance’s predictability by learning a simple model, while modelSHAP uses additive principles of feature attribution to characterize contributions to predictions (Kaur et al., 2025; Nazri and Razak, 2026; Özüpak et al., 2025; Verma et al., 2025a).

There are two reasons for this: first, the attribution patterns produced by individual XAI methods depend on the mathematical principles they are based on; second, the mathematical principles behind each method are also distinct. A single method then provides a method-specific perspective on model behavior. While complementary XAI methods can offer a variety of views on predictive evidence, it is necessary to quantify differences and similarities between them, rather than assume that multiple explanations are automatically more accurate.

### 2.4 Multi-XAI and quantitative explanation evaluation

Multiple explanation approaches can yield a complementary understanding of image features and spatial regions that affect a model’s prediction. Different mathematical principles characterize model behavior, such as gradient-based attribution, feature-attribution methods, and local surrogate explanations. Recent plant-disease studies have begun combining predictive models with various XAI methods to provide a more comprehensive explanation of classification results (Kaler and Kaur, 2025; Nazri and Razak, 2026; Rashid et al., 2025).

But creating several heatmaps that appear plausible does not mean that they are all of a high explanatory quality. Different explanation methods may be sensitive to model parameters, input transformations, perturbation procedures, and analysis choices. Hence, quantitative XAI evaluation is necessary alongside qualitative visualization. The perturbation fidelity can also assess whether the fidelity of attribution changes when the information from the model is perturbed; the transformation-based stability can characterize the consistency of the attribution patterns when a set of transformations transforms the information from the model; and the model randomization analysis can assess if the attribution patterns vary according to the learned model parameters.

Both cross-explainer analysis and cross-explaining methods can provide different perspectives, since different XAI methods do not necessarily identify the same spatial regions. Therefore, low spatial agreement does not mean one explanation is wrong, and high agreement does not mean the explanation is biologically correct. Therefore, it is important to be able to distinguish between the three properties of spatial agreement, attribution-burden agreement, and fusion sensitivity, and have all three properties reported separately. Individuals, pairs, and triplets of explainers can be evaluated for their behavior, as well as for the behavior of the fusion, to see if the fusion actually exhibits complementary behavior, not just that it is superior in general.

### 2.5 Attribution maps and disease-severity interpretation

Explainable classification differs from, but is related to, disease segmentation and estimating disease severity in plants. If pixel-level lesion annotation is available, semantic segmentation techniques can explicitly locate diseased tissue and quantify lesion or disease extent (Polly and Devi, 2024; Verma et al., 2025b; Yang et al., 2024; Zhao W, et al., 2025). Researchers typically study these pixel-level segmentation tasks using U-Net and other encoder–decoder-based architectures.

XAI attribution maps serve a different purpose. They do not segment pathological tissue; instead, they identify image regions that help a model predict the tissue. Regions with high attribution values can refer to lesion characteristics, disease-free morphology, venation boundaries, texture, color, or other discriminative visual evidence. Therefore, an attribution map may not necessarily be interpreted as being the same spatial scale as a biological extent of disease.

This difference is especially significant when there is no ground truth (GT) or lesion masks from independent experts. In this scenario, spatial measures from attribution maps are better thought of as prediction-relevant attribution measures, or relative attribution-burden measures, rather than as validated lesion localizations or quantitative disease-severity estimates. Variations in sensitivity and consensus among different measures of the attribution burden can define the behavior of these spatial summaries, but they are not necessarily biologically correct.

### 2.6 Research gap

Although significant advances have been made in deep-learning classification and explanation of plant diseases and in Agricultural AI, methodological gaps remain. While some of the above aspects have been studied in isolation, others, such as preprocessing, deep architectures, transfer learning, attention mechanisms, and XAI, have not been studied or examined in a single controlled experimental protocol, which separates model-development decisions from held-out evaluations. Dataset composition, image quality, background characteristics, class imbalance, and acquisition conditions may affect reported classification results.

Thus, it is crucial to conduct careful dataset auditing, use leakage-aware partitioning, validate datasets for appropriate preprocessing and architecture selection, and isolate data for the held-out test set. A second gap is in modeling stochastic variation. The reported performance comes from a single trained model and does not reflect variability due to random initialization and optimization. Therefore, evaluating different independent training runs could provide insight into training stability, but it does not constitute full computational reproducibility.

The third gap concerns explainability assessment. New technologies like gradient-based, feature-attribution, and local surrogate methods are also becoming a common way to study plant-disease XAI. Individual methods can produce method-specific attribution patterns, and qualitative pictorial representations are not sufficient for explanation fidelity, stability, understanding of parameter dependence, and/or cross-method agreement. Many XAI methods are now being considered for wider knowledge of plant-disease classifiers, Motivating Quantitative comparison of individual explainers, their pairwise combinations, and multi-method fusion. Although recent studies increasingly incorporate multiple XAI methods and some have introduced quantitative faithfulness assessment, a broader evaluation that jointly examines perturbation fidelity, transformation stability, model-parameter dependence, fusion sensitivity, cross-explainer spatial agreement, attribution-burden stability, and computational cost remains comparatively limited.

A fourth gap concerns interpreting spatial attribution. Attribution maps may show visual evidence that is relevant to the predictions, not lesion boundaries in biological structures. Lesion area or disease severity assessments must be quantitative, which means suitable lesion annotations (ground truth) must be obtained at the pixel level. Hence, attribution-based spatial indicators should not be used as disease severity estimates in the absence of disease severity estimates from independent experts.

Further to address these methodological gaps, AgroXAI is formulated as an integrated evaluation framework rather than as a novel backbone or a novel XAI algorithm. The framework combines dataset-integrity auditing and group-disjoint partitioning; validation-only preprocessing and architecture selection; five independently initialized training runs; fixed held-out evaluation; probability calibration and statistical uncertainty analysis; and complementary Grad-CAM++, SHAP, and LIME evaluation. The primary task is formulated as a 14-class joint species–health classification problem, while the same predictions are deterministically aggregated into seven species categories and two health-status categories for multi-level evaluation without introducing separate hierarchical classifiers. The XAI component evaluates individual, pairwise, and three-way explanations through perturbation fidelity, controlled-transformation stability, model-randomization behavior, fusion sensitivity, cross-explainer spatial agreement, attribution-burden stability, and computational cost. Attribution maps are interpreted as prediction-relevant explanations rather than validated lesion localization or disease-severity estimates.

**Table 1**. summarizes the methodological characteristics of representative plant-disease classification and explainability studies already discussed in this review. Recent studies demonstrate substantial progress in deep-learning classification, model ensembling, and the use of multiple XAI methods, with some also incorporating statistical testing or quantitative faithfulness assessment. However, the comparison indicates that the simultaneous integration of leakage-aware group-disjoint evaluation, multi-seed training-stability assessment, probability calibration, statistical uncertainty analysis, and multi-dimensional quantitative evaluation of complementary XAI methods remains limited. AgroXAI is designed to address this combined methodological gap rather than to claim novelty for the Swin Transformer backbone or the individual Grad-CAM++, SHAP, and LIME algorithms.

**Table 1:** Comparison of methodological characteristics of representative plant-disease classification and explainability studies with the proposed AgroXAI system.

Study	DL classification	Leakage-aware evaluation	Multi-seed training assessment	Probability calibration	Multiple XAI methods	Quantitative XAI evaluation	Explicit attribution ≠ lesion distinction	Principal gap relative to AgroXAI
(Nahiduzzaman et al., 2023)	PDS-CNN	NR	NR	NR	Single — SHAP	Partial — qualitative/expert-supported interpretation; no reported fidelity–stability–randomization protocol	NR	Adds leakage-aware evaluation, calibration, Multi-XAI, and quantitative XAI assessment
(Natarajan et al., 2024)	Deep CNN + customized KNN	NR	NR	NR	Grad-CAM + Occlusion Sensitivity	Partial — occlusion is perturbation-based, but a separate multi-metric XAI fidelity/stability/randomization evaluation is not reported	NR	Adds multi-seed evaluation, calibration, and broader quantitative XAI validation
(Kaur et al., 2025)	EfficientNetB3 + InceptionResNetV2 hybrid	NR	NR	NR	SHAP + LIME	NR for fidelity/stability/randomization-style validation	NR	Adds leakage-aware evaluation, calibration, and quantitative cross-explainer analysis
(Özüpak et al., 2025)	MobileNetV2 + ViT + stacking	NR	NR	NR	SHAP + LIME + Grad-CAM	NR for systematic fidelity/stability/randomization evaluation	NR	Adds multi-seed assessment, calibration, and systematic XAI validation
(Rashid et al., 2025)	Multi-architecture ensemble	NR	NR	NR	Multiple XAI/CAM-based explanations	NR for systematic fidelity/stability/randomization evaluation	NR	Adds leakage auditing, multi-seed assessment, calibration, and broader XAI evaluation
(Verma et al., 2025b)	Multiple transfer-learning architectures	NR	NR	NR	Single — Grad-CAM++	NR	NR	Extends single-XAI analysis to complementary Multi-XAI evaluation
(Nazri and Razak, 2026)	Multiple CNNs + Hybrid CNN	NR	NR	NR	SHAP + LIME + Grad-CAM	Faithfulness analysis reported	NR	Extends faithfulness analysis with leakage control, calibration, stability, randomization, and fusion analysis
Proposed AgroXAI	Swin-T-based 14-class joint classifier	Dataset audit + group-disjoint train/validation/test partitioning	Five independently initialized runs	Temperature scaling + ECE	Grad-CAM++ + SHAP + LIME; individual, pairwise, and three-way analysis	Fidelity, transformation stability, model randomization, fusion sensitivity, spatial agreement, attribution-burden stability, and runtime	Explicitly distinguishes prediction attribution from biological lesion localization/severity	Integrates leakage-aware evaluation, training stability, calibration, uncertainty analysis, and quantitative Multi-XAI assessment

## 3 Materials and methodology

The proposed AgroXAI framework combines data-integrity verification, controlled pre-processing and architecture selection, transformer-based classification, training stability analysis across multiple seeds, predictive calibration, statistical uncertainty analysis, and complementary explainable AI for multi-species plant-disease classification. The experimental workflow involves sanitizing and auditing data leakage in the datasets, performing group-disjoint data partitioning, ablations of the data preprocessing, screening the architecture, training the model, running five times with different random seeds, fixed held-out evaluation, probability calibration, and quantitative evaluation of Grad-CAM++, SHAP, LIME, and their complementary fusions. The architecture and checkpoints are selected solely from the training and validation partitions. All preprocessing, architecture-selection, and checkpoint-selection decisions are made in the training and validation partitions. These model development decisions are made without using the held-out test partition, and the held-out test partition is used only after the final configuration and checkpoints are determined. The overall workflow of the proposed AgroXAI framework, including dataset preparation, leakage-aware partitioning, model development, held-out evaluation, and Multi-XAI explainability analysis, is illustrated in **Figure 1** .

**Figure 1:**
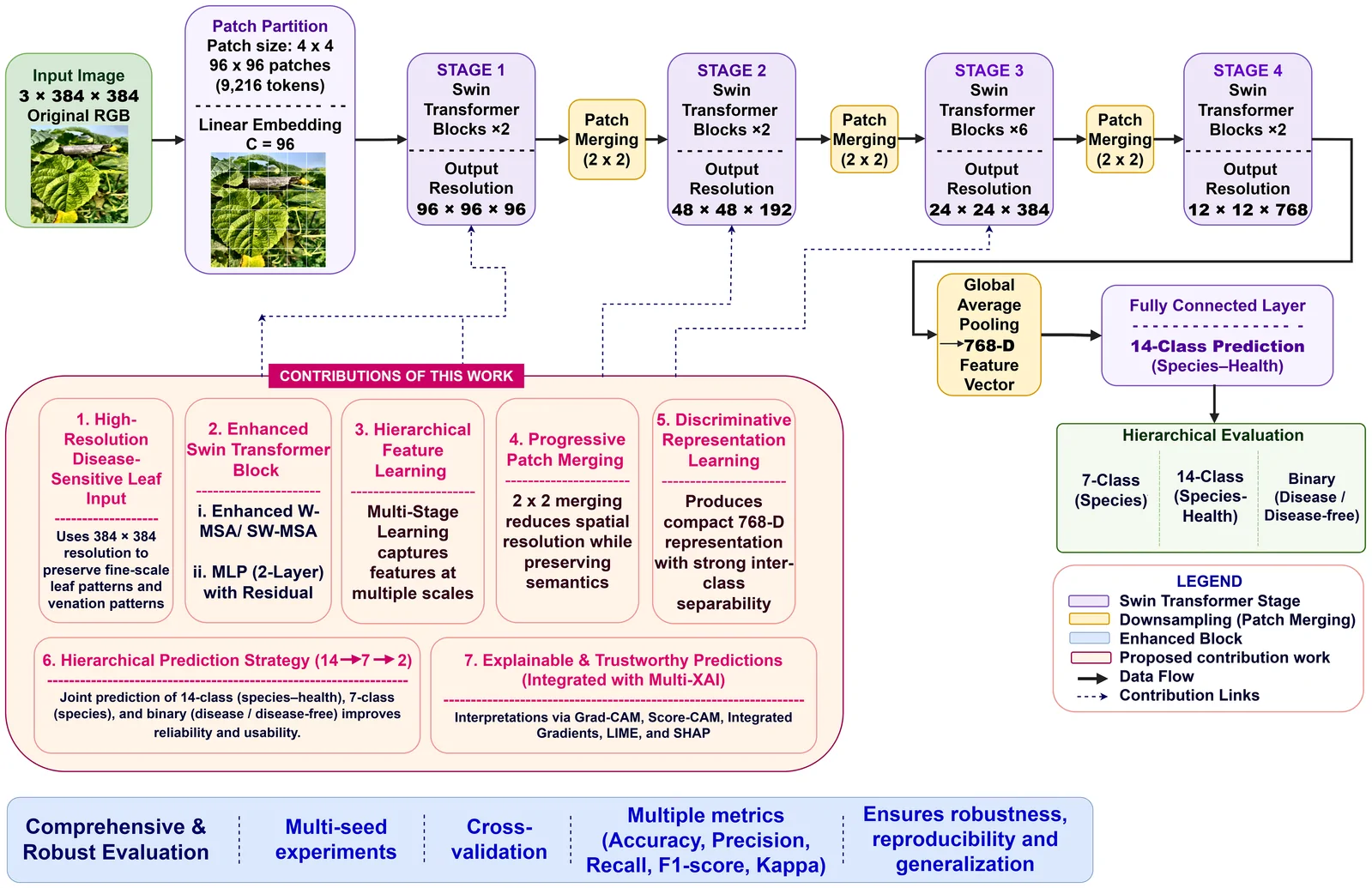
Proposed AgroX-Swin architecture and multi-level evaluation for multi-species plant disease classification.

AgroX-Swin is the classification part of the framework, based on a Swin Transformer-Tiny (Swin-T) backbone with 384 × 384 RGB input. **Figure 2** summarizes the controlled workflow: from dataset-integrity auditing and leakage-aware partitioning to model development, held-out evaluation, calibration, statistical analysis, and quantitative Multi-XAI evaluation.

**Figure 2:**
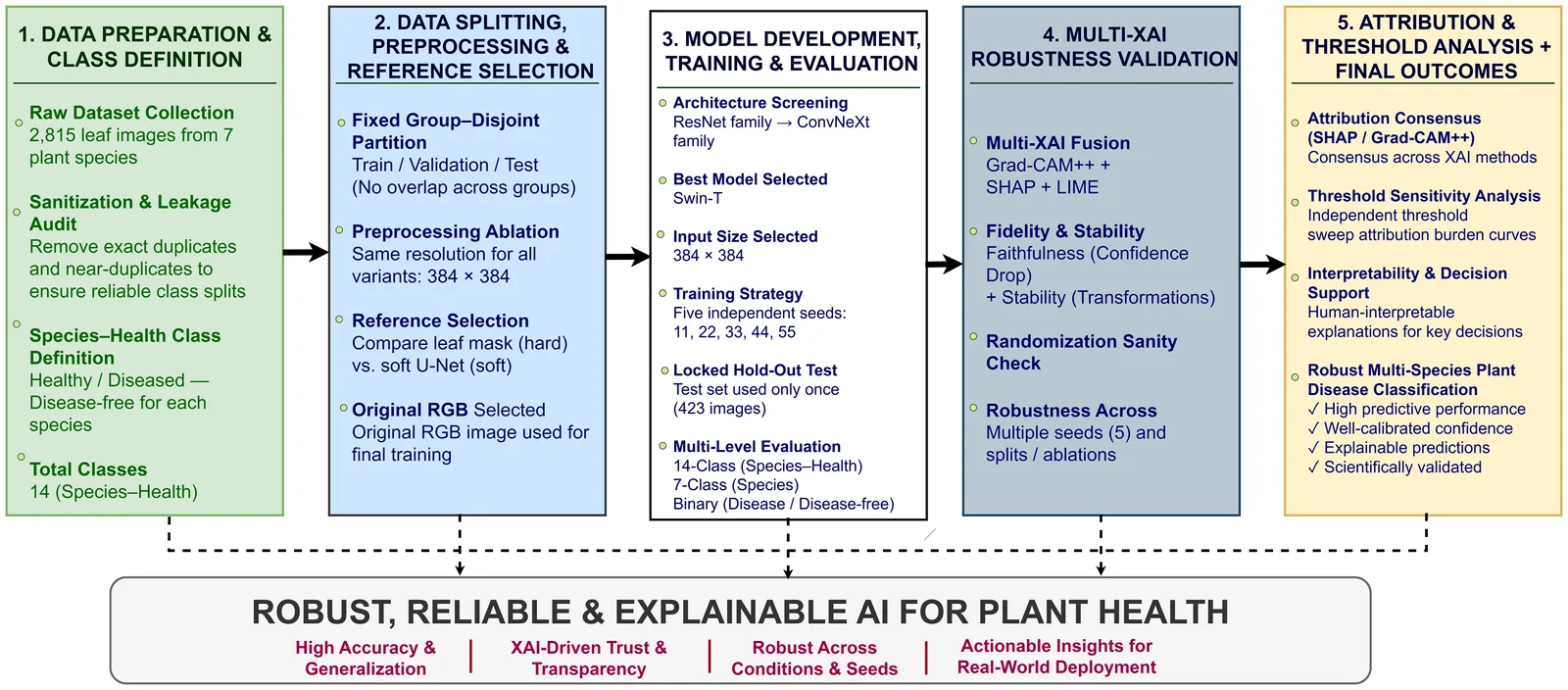
Proposed AgroXAI workflow for explainable multi-species plant disease classification.

### 3.1 Dataset description, organization, and classification task

The study uses a plant-leaf image dataset reported by (Zitu et al., 2024), which consists of 2,815 images of seven plant species and is publicly available. The dataset has 14 joint species–health classes, with each species represented in two health status classes: Disease and Disease-free. Each of these 7 species is paired with two health classes, yielding 14 final species–health classes. Therefore, the main learning problem is not 14-species classification, but 14-class classification. Each class encodes both species identity and health status; for instance, Lagenaria siceraria/Disease and Lagenaria siceraria/Disease-free are two separate joint classes of the same species.

Because the label is joint (species and health), a joint formulation is adopted to directly predict both the species label and the health label from a single classifier. This prevents a cascading species→health pipeline that might pass a mispredicted species label to the next health-status decision. Thus, the main classification goal is to classify the 14-class joint species–health task.

Two more levels are deterministically mapped from the same 14-class output to serve as complementary evaluation. First, the predictions are combined into seven species categories, regardless of health status, to test species recognition. Second, they are binned into Disease and Disease-free categories (by species) across all observations, regardless of the number of observations, to assess binary health-status discrimination. These mappings are not hierarchical or multi-stage classifiers; they are used only for multi-level evaluation.

As shown in **Table 2**, the dataset is organized into 14 joint species–health classes representing seven plant species under Disease and Disease-free conditions. The fixed training, validation, and held-out test partitions preserve representation of all classes. This distribution supports consistent evaluation of the primary 14-class classification task across the predefined experimental partitions. 

**Table 2:** Distribution of the 14 species–health classes across the fixed experimental partitions.

Species	Health status	Total	Training	Validation	Test
Seven species × two states	Disease / Disease-free	**2,815**	**1,970**	**422**	**423**

Bold values indicate values highlighted for emphasis within the corresponding table.

### 3.2 Dataset integrity audit and leakage-aware partitioning

Before model development, the dataset is subjected to a systematic integrity audit to minimize the possibility that identical or highly related images occur across model-development and evaluation partitions. Exact duplicate detection is performed using SHA-256 content hashing. Perceptual screening is additionally performed using the ImageHash implementation of perceptual hashing (phash) with hash_size = 8, corresponding to a 64-bit perceptual hash. In the implemented screening procedure, images with identical perceptual-hash values are treated as perceptual duplicate candidates, corresponding to a Hamming distance of zero. Potential source-related candidates are additionally subjected to manual review. Filename similarity alone is not treated as sufficient evidence for assigning images to the same source group.

Following the final audit and evidence-group reconstruction, the dataset contains 2,815 images assigned to 40 evidence-supported groups, all of which are singletons. No unresolved exact-duplicate groups or retained perceptual duplicate candidate groups remain in the finalized dataset. A separate manual review of 40 filename/source duplicate candidates classifies all 40 as not_same_source_group, with no confirmed or unresolved same-source group remaining.

The finalized dataset is split into approximately 70:15:15 training, validation, and held-out test sets, with final counts of 1,970, 422, and 423 images, respectively. Partition integrity is checked using image identifiers, file paths, and evidence/source-group identifiers. The training, validation, and test partitions have no overlap in image IDs, paths, or group identifiers, and each of the 14 joint species–health classes appear in each partition. These overlap checks, along with the original manuscript, confirm split sizes.

The training partition optimizes the parameters; the validation partition supports preprocessing selection, architecture comparison, checkpoint selection, and calibration development. The test partition is not used for preprocessing selection, architecture selection, hyperparameter optimization, or checkpoint selection. For the fixed partition assignments, they are carried along the experimental pipeline.

Only training images are stochastically augmented after partitioning. Validation or held-out test images are not augmented.

### 3.3 Preprocessing ablation

Foreground extraction can suppress potentially irrelevant background information, but segmentation can also alter texture, boundary, color, or structural information that may contribute to classification. Therefore, foreground segmentation is not assumed as a priori to improve classification. Three matched input representations are evaluated: Original RGB, Hard U2Net, and Soft U2Net.

Original RGB retains the complete leaf image and surrounding context without foreground segmentation.

Hard U2Net uses U2Net-derived saliency information to construct a binary foreground mask that separates the estimated leaf region from the background. Soft U2Net uses the saliency information as a continuous weighting function, attenuating rather than completely removing contextual information.

All preprocessing alternatives are generated from the same sanitized images and evaluated using the same fixed training and validation partitions. The common output resolution is 384×384 pixels, and CLAHE-based enhancement is applied consistently so that foreground treatment constitutes the principal preprocessing variable under comparison. The held-out test partition is not used to select the preprocessing strategy. The original methodology already specifies these three alternatives and their common experimental conditions.

During final model training, stochastic augmentation is applied exclusively to the training partition using RandomResizedCrop to 384×384 pixels with a scale range of 0.90–1.00 and aspect-ratio range of 0.95–1.05, random horizontal flipping with probability 0.50, random rotation within ±10°, and ColorJitter with brightness = 0.10, contrast = 0.10, and saturation = 0.08, followed by ImageNet normalization. Validation and held-out test inputs undergo deterministic preprocessing without stochastic augmentation.

### 3.4 Deep learning architecture screening and model selection

Following preprocessing selection, candidate architectures are tested on the same fixed training and validation partitions, with a fixed input representation. Architectures used in the screening process include EfficientNetV2-S + CBAM, ConvNeXt-Tiny, and Swin Transformer-Tiny (Swin-T). The main model-selection criterion is validation macro-F1, since each of the 14 joint species–health classes contribute equally to macro-averaging. No screening or architecture selection is performed on the held-out test partition, and no model is selected based on its performance.

After validation, the architecture and preprocessing are frozen for the final held-out evaluation. The final AgroX-Swin classifier is Swin-T, which takes RGB inputs of size 384 × 384. The input is divided into 4 × 4 patches and passed through four levels of Swin Transformers with different depths [2, 2, 6, 2] and feature dimensions of 96, 192, 384, and 768, respectively. The resulting representation is pooled globally and fed to a 14-class classification head.

The overall classification operation is expressed as the mathematical formulation of the proposed classification and training procedure is presented in Equations 1–4

(1) $$X_{RGB}^{384\times 384}\to \text{Swin‐T}\to z\to \text{Softmax}\to \hat{y}$$

where XRGB denotes the input RGB leaf image, $z=\left[z_{1},z_{2},\ldots ,z_{C}\right]$ represents the output logits, C = 14 represents the number of joint species–health classes, and $\hat{y}$ denotes the predicted class.

Swin-T hierarchically learns visual representations through shifted-window self-attention. The attention operation is expressed as

(2) $$\text{Attention}\left(Q,K,V\right)=\text{Softmax}\left(\frac{QK^{T}}{\sqrt{d_{k}}}+B\right)V$$

where Q, K, and V represent the query, key, and value matrices, respectively; dk represents the dimensionality of the key vectors, and B represents relative positional bias.

The class probabilities are subsequently obtained through the Softmax operation:

(3) $$p_{c}=\frac{\exp\left(z_{c}\right)}{\sum_{j=1}^{C}\exp\left(z_{j}\right)},\;c=1,\ldots ,C$$

where pc denotes the predicted probability for the cth species–health class.

### 3.5 Multi-seed training, held-out evaluation, and error analysis

The best-configured AgroX-Swin is trained repeatedly with 5 random initializations (seeds 11, 22, 33, 44, and 55) and stochastic optimization. These experiments are not computational reproducibility tests; they are a multi-seed training-stability test. Checkpoint selection is based only on macro-F1 for each run. These five independent seeds, along with checkpoint selection by the old methodology, are already in use.

In cases where there is class imbalance during optimization, it is taken care of by employing a class-weighted cross-entropy objective:

(4) $${ℒ}_{WCE}=-\frac{1}{N}\sum_{i=1}^{N}w_{y_{i}}\log p_{i,y_{i}}$$

where N denotes the number of training samples, yi represents the ground-truth class for sample i, $p_{i,y_{i}}$ represents the probability assigned to its correct class, and $w_{y_{i}}$ denotes the corresponding class weight.

Once the five checkpoints are established, each model is then tested once on the held-out test partition of 423 images. The architecture, the preprocessing, and the hyperparameter or checkpoint-selection decision are never changed after seeing the test results.

Accuracy, class-wise precision, Recall (sensitivity), Specificity, F1 score, False negative rate (FNR), and False positive rate (FPR) are used to evaluate performance on the primary 14-class task. Macro-F1 is used as the primary summary measure because it does not weight by the number of joint classes.

Performance is assessed using accuracy, precision, recall, and F1-score. For each class, precision and recall are defined as the classification performance measures used in this study are defined in Equations 5–7.

(5) $$\text{Precision}=\frac{TP}{TP+FP},\;\text{Recall}=\frac{TP}{TP+FN}$$

while the F1-score is calculated as

(6) $$F1=2\frac{\text{Precision}\times \text{Recall}}{\text{Precision}+\text{Recall}}$$

Because the primary classification problem contains 14 species–health classes, macro-F1 is used as a principal performance measure:

(7) $$F1_{\text{macro}}=\frac{1}{C}\sum_{c=1}^{C}F1_{c},\;C=14$$

where F1c represents the F1-score for the cth species–health class.

These 14 classes are deterministically combined into two health-status classes and seven species classes. For binary classification (Healthy vs. Diseased), Disease is the positive class, and the counts for TP, FN, FP, and TN are used to calculate the metrics above. Error patterns that discriminate between diseased and disease-free individuals within each species are also analyzed to describe species-specific health-status discrimination further.

A five-model probability ensemble is created from the probability outputs of the five independently trained models, class-wise. Ensemble performance is computed on the same images used for the held-out test and is reported separately from individual-model performance.

### 3.6 Prediction calibration

Classification discrimination and predictive calibration are tested independently. The “Expected Calibration Error” (ECE), “negative log-likelihood” (NLL), “multiclass Brier score”, the reliability diagram, and confidence distributions are used to assess calibration. These calibration measures are already identified in the original methodology, which outlines the validation-fitted temperature scaling.

ECE quantifies the discrepancy between model confidence and empirical accuracy and is calculated as the formulations for expected calibration error and temperature-scaled class probabilities are given in Equations 8 and 9, espectively.

(8) $$ECE=\sum_{m=1}^{M}\frac{\left|B_{m}\right|}{N}\left|acc\left(B_{m}\right)-conf\left(B_{m}\right)\right|$$

where *B_m_* denotes the predictions belonging to confidence bin *m*, *acc* (*B_m_*) represents their empirical accuracy, *conf* (*B_m_*) represents their mean predicted confidence, and *N* represents the total number of evaluated samples.

*Post-hoc* temperature scaling is additionally applied to improve probability calibration without modifying the underlying classification model. The calibrated class probability is given by

(9) $$p_{c}^{\left(T\right)}=\frac{\exp\left(z_{c}/T\right)}{\sum_{j=1}^{C}\exp\left(z_{j}/T\right)}$$

where *T* > 0 represents the temperature parameter.

The held-out test predictions are used the same way as the validation-set predictions to determine the temperature parameter, then it is applied to the held-out test predictions. Test tags are not used to measure the temperature. The calibration results are given separately for the individual model and the 5-model probability ensemble.

The probabilities assigned to the seven Disease joint classes are added together to give the probability of Disease, and the probabilities assigned to the seven Disease-free joint classes are added together to give the probability of Disease-free.

Avoid putting the representative seed-44 temperature into the ensemble. Ensemble temperature scaling is reported; then the temperature must be adjusted separately, and validated ensemble probabilities are used.

### 3.7 Multi-XAI explainability and fusion

AgroXAI uses three complementary *post-hoc* explanation methods: Grad-CAM++, SHAP, and LIME. In contrast, Grad-CAM++ generates spatial attributions in a gradient-based, class-discriminative, and spatially localized manner; SHAP offers feature-attribution estimates based on Shapley-value principles; and LIME distills local model behavior by adding perturbations and fitting an interpretable surrogate model.

Because these techniques employ different explanation principles and generate attribution values at different numerical scales, each attribution map is normalized before fusion the normalization of individual attribution maps and their equal-weight Multi-XAI fusion are defined in Equations 10 and Equation 11, respectively:

(10) $${\hat{M}}_{k}\left(x,y\right)=\frac{M_{k}\left(x,y\right)-\min\left(M_{k}\right)}{\max\left(M_{k}\right)-\min\left(M_{k}\right)+\epsilon },$$

where Mk represents the attribution map generated by explanation method k, ${\hat{M}}_{k}$ denotes its normalized representation, and ϵ is a small numerical constant.

The three normalized explanation maps are subsequently integrated using equal-weight Multi-XAI fusion:

(11) $$M_{\text{fusion}}=\frac{{\hat{M}}_{\text{GC}++}+{\hat{M}}_{\text{SHAP}}+{\hat{M}}_{\text{LIME}}}{3}$$

Equal weighting is pre-specified to prevent data snooping and optimize the explainers’ weights on a held-out evaluation set. An unequal-weight fusion that is not optimized or learned is also considered, as a sensitivity analysis to check whether the attribution representation is strongly correlated with the fusion weights.

Detailed XAI evaluation is performed on a representative model chosen based on validation macro-F1 from the five models already finalized for the model family. For XAI analysis, test performance is not considered the model-selection criterion.

### 3.8 Quantitative evaluation of explainability

The quantitative XAI evaluation is performed on a pre-defined subset of 42 test images (3 from each of the 14 joint species–health classes) taken from the test set, which is class-balanced. The maximum balanced subset allowed (according to the smallest class support in the held-out partition) is three images per class. Images are algorithmically selected with a fixed seed (2026), not according to the visual appearance of the image explanations.

The analysis evaluates prediction relevance and explanation behavior. It does not constitute biological validation of lesion localization.

#### 3.8.1 Perturbation-based explanation fidelity analysis

The fidelity of the perturbative methods is compared with the other four methods (Grad-CAM++, SHAP, the Three Pairwise fusions, and the Three-way Multi-XAI fusion). For every explanation map, predefined percentages of 5%, 10%, 20%, and 30% of the pixels in a given area of the leaf are selected based on max, min, or random selection.

The selected pixels are perturbed by setting the model inputs (all three channels) at those spatial coordinates to zero. Random-region controls are selected 10 times for each image and perturbation fraction, and the confidence changes are averaged. The target class matches the original unperturbed image during the corresponding perturbation comparisons, where the class is predicted from the original unperturbed image.

Grad-CAM++ fidelity is examined by perturbing highly attributed regions and measuring the corresponding reduction in confidence for the originally predicted class. The perturbation-based explanation fidelity measure is calculated using Equation 12.

(12) $$\text{Δ}P=P_{c}\left(X\right)-P_{c}\left(X_{\text{perturbed}}\right)$$

where *Pc(X)* denotes the original predicted confidence for class *c*, and *Pc*(*X*_perturbed_) represents the confidence obtained after perturbing highly attributed regions.

A positive Δ*P* indicates that removing or perturbing the highlighted region reduces confidence in the original prediction, thereby providing evidence that the explanation identifies prediction-relevant information.

Importantly, a confidence reduction demonstrates prediction relevance, not biological lesion-localization accuracy.

#### 3.8.2 Transformation-based explanation stability analysis

Transformation-based stability is evaluated under four predefined mild input transformations: brightness adjustment (± 0.03 in normalized RGB-equivalent intensity), Gaussian noise (σ = 0.01 in normalized RGB-equivalent intensity), translation by +4 pixels, and horizontal flipping. Spatially transformed attribution maps are inverse-aligned to the original coordinate system before comparison.

Stability for each explainer and fusion method is quantified using rank-based attribution similarity and attribution-burden stability. Prediction preservation is also measured to see whether the same class is predicted after each controlled transformation.

For thresholded attribution regions, IoU is calculated as the transformation-based stability of the explanation maps is quantified using Equation 13.

(13) $$IoU=\frac{\left|M_{o}\cap M_{t}\right|}{\left|M_{o}\cup M_{t}\right|},$$

where *M_o_* represents the attribution region from the original image and *M_t_* represents the aligned attribution region obtained after transformation.

The transformations are carefully restricted to mild perturbations and are used to assess controlled explanation stability. They are not taken as a sign of robustness to acquisition conditions in the field, geographic variation, unseen devices, or external datasets.

#### 3.8.3 Model-randomization sanity testing

To test whether Grad-CAM++ attribution maps are random with respect to the model parameters, model-randomization sanity testing is performed. A set of 30 held-out test images is used to evaluate the representative trained AgroX-Swin model (seed 44). The fully trained model generates maps using Grad-CAM++, and they are stored as reference explanations.

Three different randomizations are performed: (1) reinitialize the classification head; (2) reinitialize the final Swin feature stage; and (3) reinitialize the final two Swin feature stages. A new instance of the trained representative model is loaded for each condition, and native parameter-resetting operations are used to reset the corresponding modules. Therefore, the conditions are tested separately, not as a sequence of randomizations.

Each randomized model generates a map, and the maps are compared with the reference maps on the same images (Grad-CAM++ maps). Pearson correlation, intersection-over-union of highly attributed regions, and mean absolute CAM difference are used to quantify parameter dependence. This is achieved by showing low correlation and overlap after parameter randomization, indicating that the explanation relies on parameters learned by the model.

The results of the parameter-sensitivity sanity check in the randomization experiment are not meant to imply biological correctness or lesion-localization accuracy but are interpreted as such.

### 3.9 Cross-explainer agreement, fusion sensitivity, and attribution-burden analysis

Cross-explainer agreement is assessed independently of attribution-burden agreement. The rank-based spatial agreement of highly attributed regions is computed to measure pairwise spatial agreement between Grad-CAM++, SHAP, and LIME. These measures represent the number of similar spatial evidence identified, not biological agreement with lesion location.

The sensitivity of the fusion is compared with an alternative unequal-weight fusion using map-level association and top-attribution overlap, using prespecified equal-weight three-way fusion in both cases as benchmarks. This analysis checks whether the selected fusion weights are highly sensitive to the Multi-XAI representation.

Attribution-burden analysis characterizes the relative size of prediction-relevant attribution within each estimated leaf region.

For a threshold, the proportion of the estimated leaf region containing attribution values greater than or equal to the threshold is defined as the cross-explainer spatial agreement is quantified using Equation 14.

(14) $$A_{\text{XAI}}\left(\tau \right)=100\times \frac{\sum_{p\in L}I\left[M_{\text{fusion}}\left(p\right)\geq \tau \right]}{\left|L\right|}$$

where L represents the estimated leaf region, |L| denotes the number of pixels within the leaf region, p represents an individual pixel, and I[·] denotes the indicator function.

Because the estimated affected proportion can vary depending on the selected threshold, a threshold-independent attribution-burden measure is additionally calculated the attribution-burden measure is calculated using Equation 15:

(15) $$B_{\text{mean}}=\frac{1}{\left|L\right|}\sum_{p\in L}M_{\text{fusion}}\left(p\right)$$

Attribution-burden measures are not measures of biological disease severity; they are quantities explored during modeling. Lesion masks and ground truth for disease severity were not available for this analysis. The lesion maps and their spatial summaries therefore do not represent confirmed lesion boundaries, lesion percentages, or quantitative estimates of disease severity.

This distinction matters, especially since the old manuscript treats agreement and burden on spatial XAI as a single property. They do not. The manuscript reports pairwise spatial agreement of about 0.27, and burden-measure consensus of 0.9418.

### 3.10 Statistical validation and reproducibility analysis

The experimental unit defines the statistical analyses. The training run is the experimental unit (n = 5), with 5 independently initialized training runs. The results of the 5 runs are summarized with the arithmetic mean, sample standard deviation, and a 95% confidence interval based on a Student’s t-distribution.

For a performance metric obtained across independent runs, the mean and standard deviation are calculated as the corresponding attribution-burden agreement/stability measure is quantified using Equation 16.

(16) $$\bar{x}=\frac{1}{n}\sum_{i=1}^{n}x_{i},\;SD=\sqrt{\frac{\sum_{i=1}^{n}{\left(x_{i}-\bar{x}\right)}^{2}}{n-1}}$$

For the five-seed experiments (n = 5), the 95% confidence interval is calculated using the Student’s t-distribution:

(17) $$CI_{95\%}=\bar{x}\pm t_{0.975,n-1}\frac{SD}{\sqrt{n}}$$

Given that only five independent training runs are available, interpret the interval with caution; it estimates the variability across training runs, not complete computational reproducibility.

The held-out image is the experimental unit for comparing the correctness of the five classifiers trained independently on the held-out image, and each classifier yields repeated binary correctness indicators. Cochran’s Q test tests the null hypothesis that the marginal probabilities of correct classification are equal. A non-significant result does not mean the classifiers are identical, or that the result will be reproduced; it merely means that there is not enough evidence to conclude that the marginal correctness rates are different.

Pairwise comparisons between the classifiers are conducted (where applicable), for which the same held-out images are used, and Holm correction for the predetermined family of multiple comparisons is used.

To measure finite-sample uncertainty in ensemble performance, images from the held-out test set are resampled using bootstrapping. The bootstrap analysis uses 5000 bootstraps and random seed 2026. This resulting confidence interval reflects uncertainty due to the fixed held-out data set and fixed model predictions. Still, it does not measure uncertainty about training seeds or on independently collected/held out data sets.

In quantitative XAI comparisons, a balanced subset of 42 predetermined images is used, with each image as the experimental unit. Matched explanations created for the same image are compared by statistical methods, paired when only two hypotheses are tested; or Holm-adjusted when several prespecified hypotheses are tested.

### 3.11 Implementation and computational details

All experiments are done in Python with PyTorch and related computer-vision and scientific-computing libraries. The last classifier adopts the ImageNet pre-trained Swin Transformer-Tiny network, which requires 384 × 384 inputs. The final training runs 11, 22, 33, 44, and 55 are independently trained using random seeds. The model is trained for up to 15 epochs, using checkpoint selection and early stopping based on macro-F1 on the validation set. First, the backbone is frozen, then unfrozen for fine-tuning after initial training. The training records show the 15-epochschedule, checkpointing, backbone unfreezing after training, and early stopping.

Report the computational complexity separately for each of the following: trainable parameter count, model size, FLOPs/MACs at 384×384 resolution, inference latency, throughput, and peak GPU memory (if applicable) for a single AgroX-Swin classifier and the five-model ensemble. Please also report the training time for the last classifier.

For XAI analysis, computational time is reported separately for individual and fused explanations. The measured mean runtimes are approximately 0.0834 s/image for Grad-CAM++, 5.1638 s/image for SHAP, 13.1000 s/image for LIME, 5.2513 s/image for Grad-CAM++ + SHAP, 13.1874 s/image for Grad-CAM++ + LIME, 18.2678 s/image for SHAP + LIME, and 18.3512 s/image for the three-way Multi-XAI analysis.

## 4 Experimental results and discussion

Model development is separated from final evaluation and followed by an experimental evaluation. The 423-image held-out test partition is used only at the end, when the final configuration and checkpoints for the AgroX-Swin model are determined. Meanwhile, all preprocessing and architecture decisions are made from the training and validation partitions. The final classifier is trained on original RGB data (384x384) and tested across five training runs initialized with seeds 1, 22, 33, 44, and 55. Performance is compared at the primary species–health class level (14 classes) and at the species–binary Disease/Disease-free level (7 classes) using deterministic aggregation of the same predictions. In addition, predictive calibration, statistical uncertainty, error structure, and the quantitative behavior of Grad-CAM++, SHAP, LIME, and their complementary combinations are accounted for.

### 4.1 Preprocessing and architecture selection

Preprocessing is ablated by comparing representations from the Original RGB image, Hard U²Net, and Soft U²Net, all trained and validated on the same partitions. Original RGB is the best alternative in terms of validation performance and remains included for more in-depth analysis of possible architectures. Compared with using the full RGB image, U²Net-based foreground processing does not improve foreground validation accuracy. This finding suggests that while background information can be removed, it is not always helpful with this data and that some information such as boundary, texture, color, or context information may be useful in the original image but lead to discrimination.

**Figure 3** shows the three forms of representation – Original RGB, Hard U²Net and Soft U²Net – that were evaluated. In this work, the U²Net saliency map is used only as an intermediate representation to create the Hard U²Net and Soft U²Net variants. It is not used directly as an input for evaluation as an independent classifier.

**Figure 3:**
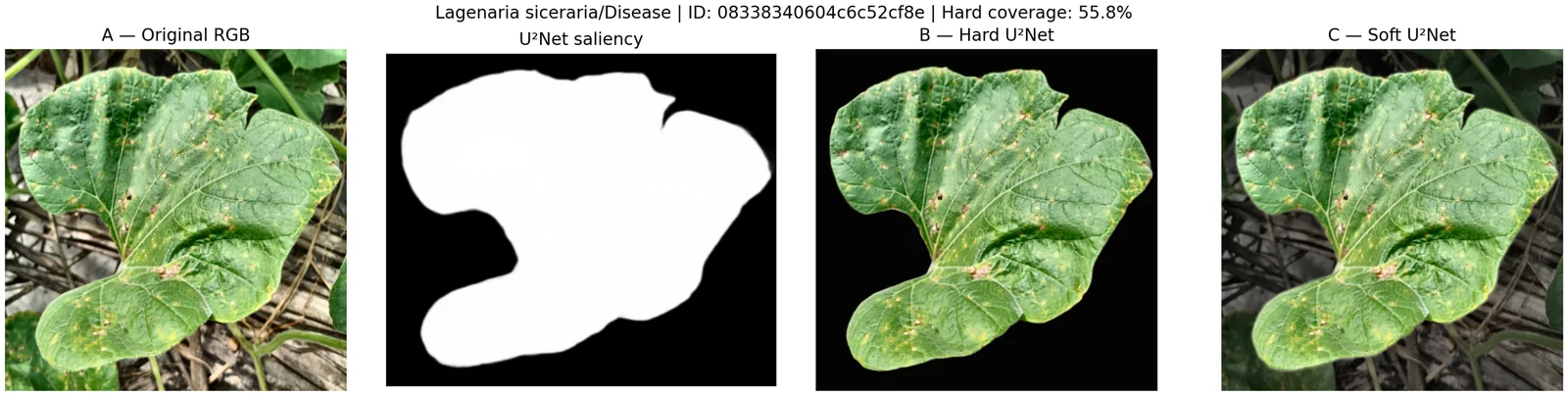
Preprocessing ablation and representative input generation.

After preprocessing selection, the performance of EfficientNetV2-S + CBAM, ConvNeXt-Tiny, and Swin-Tiny is compared using a fixed training and validation protocol. Among the candidates evaluated, Swin-Tiny has the best validation macro-F1, and it is thus chosen for further model development. This outcome is unexpected only because of the comparison between the architectures tested in this study, and it does not imply that Swin-T is always—and more generally, that it is always more effective than—other plant-disease classifiers. This test partition selection does not include the held-out test partition. The current manuscript already reports a comparison of these three architectures under the same validation protocol.

The following architecture-screening results are represented independently in **Figure 4**, where validation Macro-F1 of the proposed architectures and the final choice of Swin-T for further modeling are displayed.

**Figure 4:**
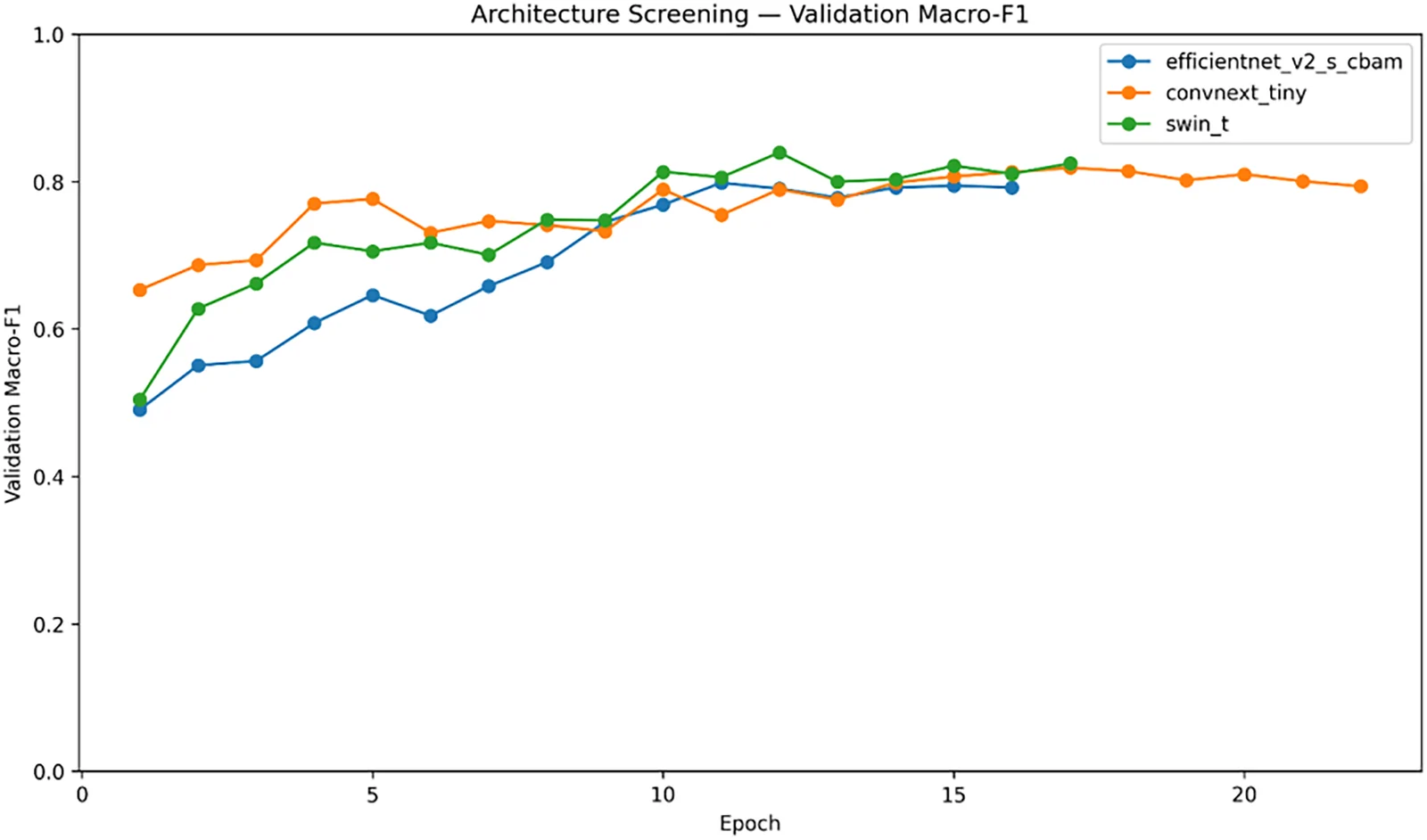
Architecture screening based on validation macro-F1.

Because none of the alternative configurations provides a meaningful validation improvement over E0, the baseline configuration is retained. Similarly, test-time augmentation does not improve validation performance, with macro-F1 values of approximately 0.8473 without TTA and 0.8449 with TTA. Consequently, the final locked configuration is Original RGB + Swin-T, 384 × 384, E0 baseline, without TTA.

As illustrated in **Figure 5**, both enhanced configurations attain close-to-competitive validation Macro-F1 values. Still, neither configuration shows a consistently significant improvement over the baseline configuration compared to the others, making it too complex to be a worthwhile avenue for exploration. Thus, the validated Swin-T backbone with the baseline classification configuration is retained to constitute the final AgroX-Swin model for multi-seed training and locked held-out evaluation.

**Figure 5:**
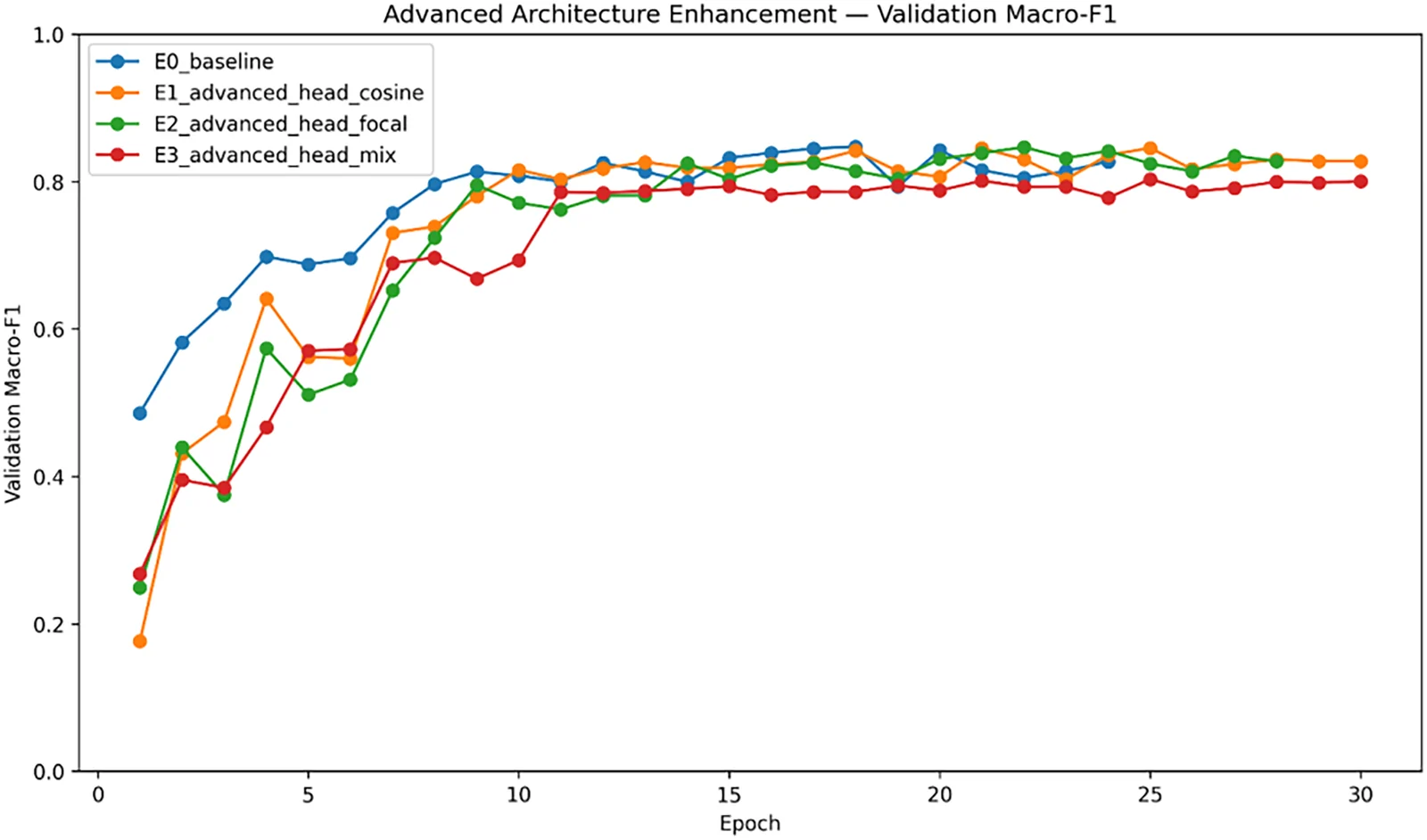
Advanced architecture-enhancement analysis based on validation macro-F1.

The validation-based experimental selection process is summarized in **Table 3**. Across the evaluated preprocessing strategies and candidate architectures, Original RGB input and Swin-Tiny are retained based on validation performance, with 384 × 384 selected as the final input resolution. Accordingly, Original RGB + Swin-T at 384 × 384 is locked as the final AgroX-Swin configuration before held-out test evaluation.

**Table 3:** Validation-based selection of the final AgroX-Swin configuration.

Experimental stage	Configurations evaluated	Selected configuration
Preprocessing	Original RGB, Hard U²Net, Soft U²Net	Original RGB
Architecture	EfficientNetV2-S + CBAM, ConvNeXt-Tiny, Swin-Tiny	Swin-Tiny
Input resolution	Validation-based configuration	384 × 384
Final model	Locked before test evaluation	Original RGB + Swin-T 384 × 384

### 4.2 Multi-seed training stability, multi-level performance, and error analysis

The AgroX-Swin configuration is trained with five random seeds after the final configuration. The accuracy of the five left-out evaluations is 87.19% on average, and the macro-F1 score is 0.8300 ± 0.0122 on average. A 95% Student’s t-confidence interval for the mean macro-F1 is: (0.8148, 0.8451). The results reflect variability among five different initializations and show relatively small variation between the runs observed. Because of the limited number of runs (only five), take the interval with a grain of salt as indicative of training-run variability, not computational reproducibility.

On the held-out test partition, the 5 trained models with the probability-level ensemble are 87.94% accurate and 0.8355 macro-F1. The ensemble gain of macro-F1 is around 0.0055, and the gain of accuracy is around 0.75 percentage points compared to the five-run mean. The gain is small and should be interpreted as a modest descriptive gain of the ensemble, or a paired significance analysis should be performed first.

This matters for several reasons, but most importantly, the current manuscript claims that the ensemble “eliminates” errors and suggests a greater improvement than the data show.

The previous 14-class ensemble predictions are deterministically combined into predictions for seven plant species and two health-status classes. Species-level accuracy is 99.29%, meaning the model correctly identifies most held-out images by species. This is a species-recognition measure only and does not indicate disease-detection accuracy. The accuracy of the binary Disease/Disease-free is 88.42%, and the F1 score for the diseased class is 0.9160. These results show that the model can discriminate between Disease and Disease-free status. The final held-out performance of AgroXAI across the five independent training seeds and the probability-level ensemble is summarized in **Table 4**.

**Table 4:** Final held-out performance of AgroXAI.

Evaluation	Performance
Mean five-seed test accuracy	87.19%
Mean five-seed macro-F1	0.8300 ± 0.0122
95% CI of five-seed macro-F1	0.8148–0.8451
Ensemble 14-class accuracy	87.94%
Ensemble 14-class macro-F1	0.8355
7-class species accuracy	99.29%
Binary health-status accuracy	88.42%
Diseased-class F1-score	0.9160

The difference between species-level accuracy (99.29%) and binary health-status accuracy (88.42%) indicates that most classification errors are not attributable to species identity. Rather, the more difficult component of the joint task is discrimination between Disease and Disease-free states within species. However, the species metric is interpreted only as species-recognition performance and not as evidence of disease recognition.

Seeds 11, 22, 33, 44, and 55 are used to train 5 different AgroX-Swin models, and the results of Accuracy and Macro-F1 are returned. The five runs give a mean accuracy of 87.19% and a mean Macro-F1 of 0.8300, while the runs using ensemble aggregation with probability-level get 87.94% accuracy and 0.8355 Macro-F1, showing the observed variation in held-out performance across the five independently initialized training runs.

As shown in **Table 5**, the five-model AgroX-Swin ensemble demonstrates varying class-wise performance across the 14 species–health categories. The error analysis reports precision, sensitivity, specificity, F1-score, false-negative rate (FNR), and false-positive rate (FPR) for each class. These results provide a more detailed view of class-specific errors beyond the overall held-out classification performance.

**Table 5:** Held-out class-wise error analysis of the five-model AgroX-Swin.

Species–health class	Support	Precision	Recall/sensitivity	Specificity	F1-score	FNR	FPR
*Lagenaria siceraria*/Disease	59	0.9167	0.9322	0.9863	0.9244	0.0678	0.0137
*Lagenaria siceraria*/Disease-free	22	0.8095	0.7727	0.9900	0.7907	0.2273	0.0100
*Luffa cylindrica* L/Disease	21	0.9130	**1.0000**	0.9950	0.9545	**0.0000**	0.0050
*Luffa cylindrica* L/Disease-free	7	0.8333	0.7143	0.9976	0.7692	0.2857	0.0024
*Momordica charantia*/Disease	42	0.9756	0.9524	0.9974	0.9639	0.0476	0.0026
*Momordica charantia*/Disease-free	3	0.5000	0.6667	0.9952	0.5714	0.3333	0.0048
*Momordica dioica*/Disease	53	0.9783	0.8491	0.9973	0.9091	0.1509	0.0027
*Momordica dioica*/Disease-free	28	0.7500	0.9643	0.9772	0.8437	0.0357	0.0228
*Phaseolus vulgaris* L/Disease	47	0.9773	0.9149	0.9973	0.9451	0.0851	0.0027
*Phaseolus vulgaris* L/Disease-free	31	0.9091	0.9677	0.9923	0.9375	0.0323	0.0077
*Solanum melongena* L/Disease	30	0.9655	0.9333	0.9975	0.9492	0.0667	0.0025
*Solanum melongena* L/Disease-free	5	0.7500	0.6000	0.9976	0.6667	0.4000	0.0024
*Vigna unguiculata* subsp. *sesquipedalis*/Disease	48	0.8500	0.7083	0.9840	0.7727	0.2917	0.0160
*Vigna unguiculata* subsp. *sesquipedalis*/Disease-free	27	0.6111	0.8148	0.9646	0.6984	0.1852	0.0354

Bold values indicate values highlighted for emphasis within the corresponding table.

As shown in **Table 6**, the binary Disease/Disease-free analysis provides a focused assessment of health-status discrimination independent of plant species. The table reports the confusion-matrix counts together with precision, sensitivity, specificity, F1-score, FNR, and FPR for both health-status categories. This analysis provides a clearer characterization of false-negative and false-positive errors in the binary health-status task.

**Table 6:** Binary disease/disease-free error analysis.

Health-status class	Support	TP	FN	FP	TN	Precision	Sensitivity/recall	Specificity	F1-score	FNR	FPR
Disease	300	268	32	16	107	0.9437	0.8933	0.8699	0.9178	0.1067	0.1301
Disease-free	123	107	16	32	268	0.7698	0.8699	0.8933	0.8168	0.1301	0.1067

In a disease-screening context, false-negative Disease predictions are particularly important to report because they correspond to diseased samples classified as Disease-free. The present analysis therefore reports FNR explicitly together with sensitivity and specificity, rather than relying only on overall accuracy.

Each row is a value of 100% for the actual species–health class, and each column is a predicted class. In the ensemble, the true positives of the test images are 372 with an overall accuracy of 87.94%. The majority of errors are strong diagonal responses, and the rest are mainly due to the confusion between Disease and Disease-free categories for each species.

The most interesting observation seen here is that Luffa cylindrica L/Disease achieves the highest class-wise recall (100%) in this test set, while some Disease-free show a lower recall. This confirms our previous interpretation that the discrimination of health states is more difficult than that of species recognition.

**Figure 6** displays the result of the 5-seed test and the ensemble result, and the normalized 14-class ensemble confusion matrix can be displayed in **Figure 7**.

**Figure 6:**
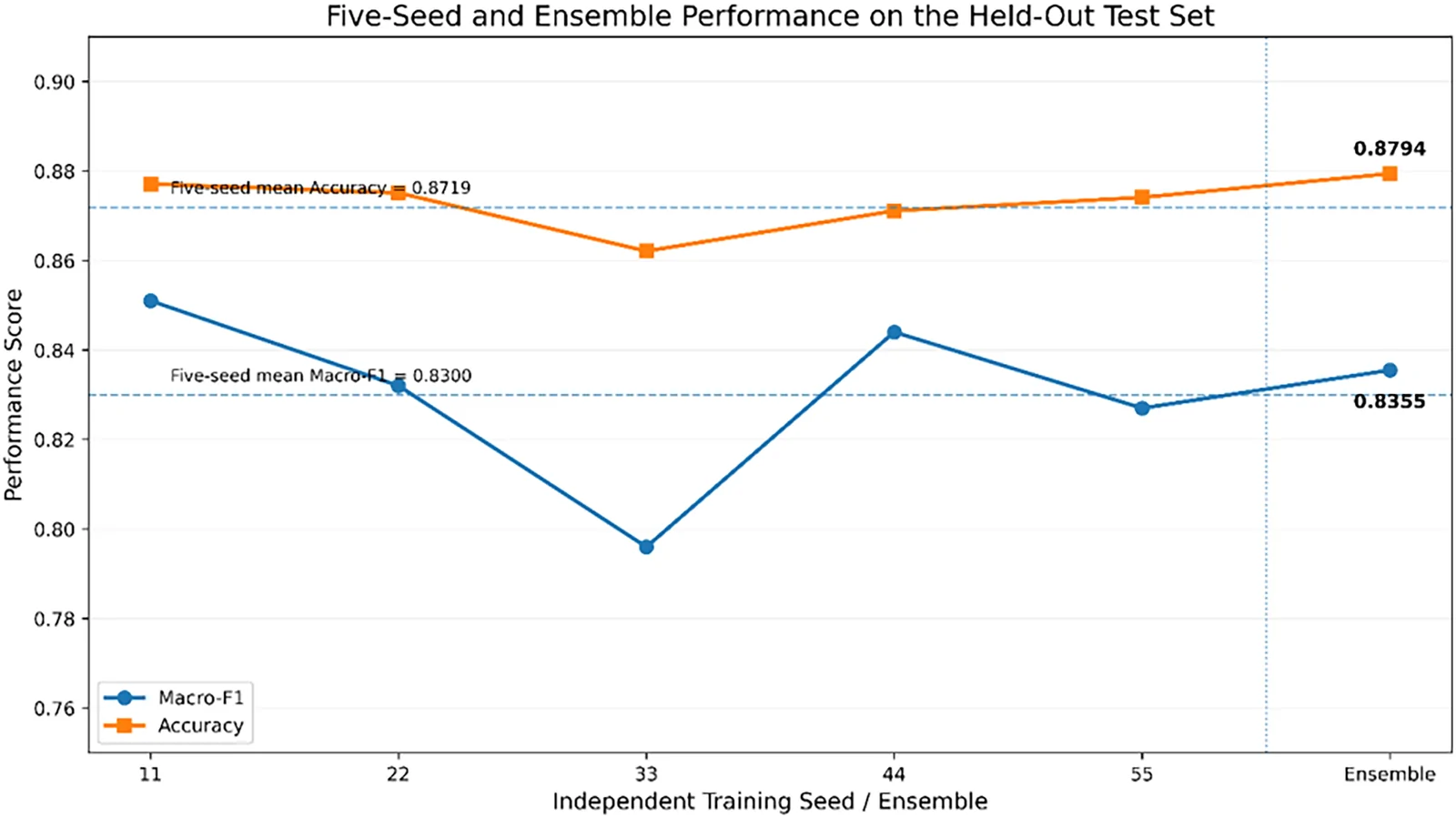
Five-seed and ensemble performance on the locked held-out test set.

**Figure 7:**
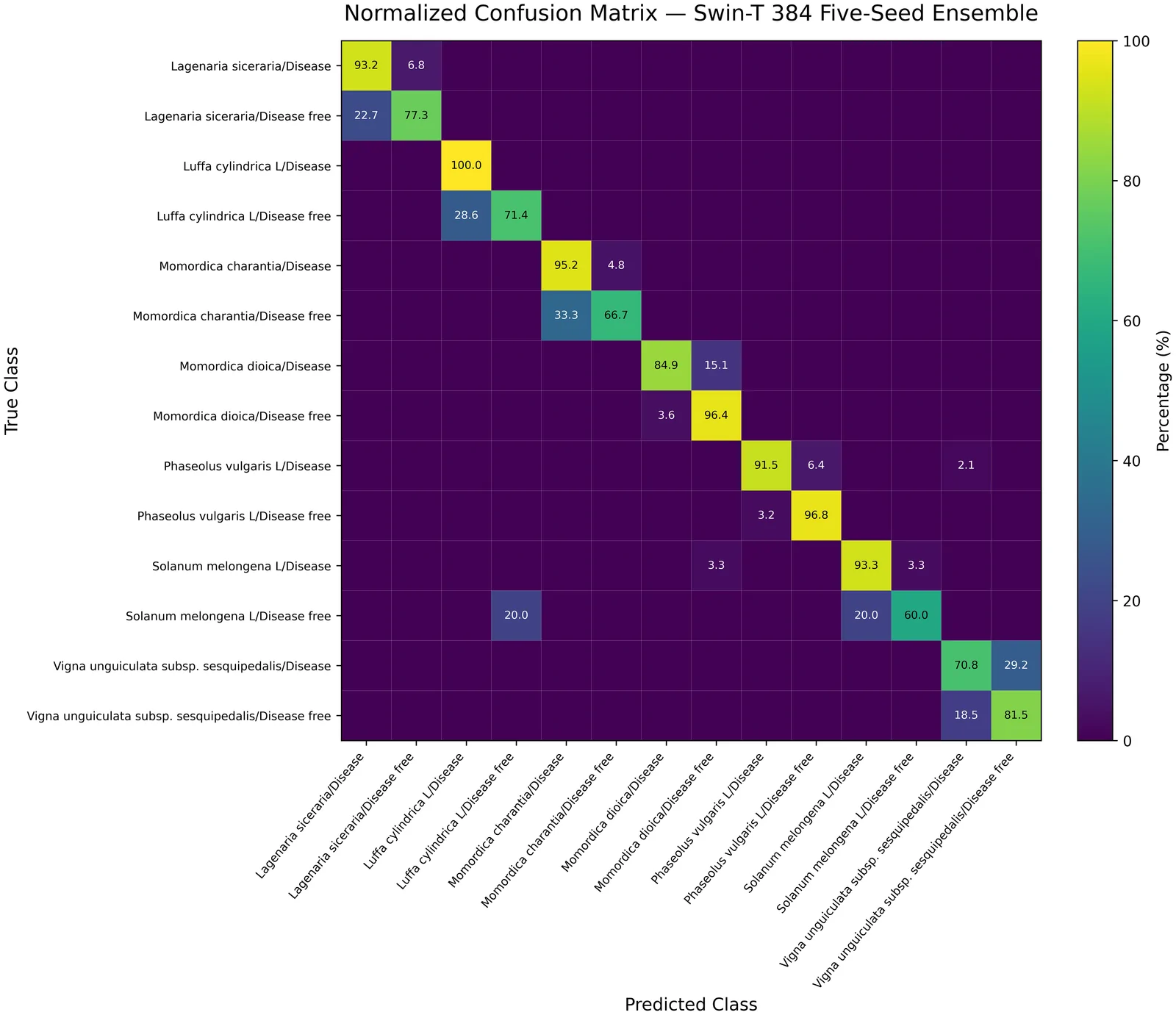
Normalized confusion matrix of the five-model ArgoX-Swin ensemble on the locked 423-image held-out test set.

### 4.3 Predictive calibration and statistical uncertainty

The ECE for the uncalibrated representative seed-44 model is 0.0792, while the ECE for the uncalibrated 5-model probability ensemble is 0.0912. The held-out ECE of the representative model decreases by about 60.9% with validation-fitted temperature scaling, from 0.0792 to 0.0310. The fitted temperature is T = 0.7794. This improvement reflects better probability calibration and does not necessarily indicate better classification accuracy, as it results from temperature adjustment. The above ECE values are already reported in the current manuscript along with the fitted temperature.

For the ensemble macro-F1 of 0.8355, 5,000 bootstrap resamples yield a 95% confidence interval of 0.7614–0.8857. The width of the interval is about 0.1243, implying that there is nontrivial uncertainty in the macro-F1 estimate for finite samples. Macro-F1 assigns equal importance to each of the 14 classes, and some held-out classes have relatively few samples, so the number of samples per class can vary substantially across bootstrap resamples, leading to large differences in class-specific F1 scores. The interval is thus an uncertainty under the assumption of a fixed held-out sample and fixed ensemble predictions. It does not capture uncertainty from the training seed or an external dataset.

The calibration and statistical reliability of the final AgroXAI model are summarized in **Table 7**, including ECE improvement after temperature scaling and statistical consistency across independent runs.

**Table 7:** Calibration and statistical analysis.

Measure	Result
Ensemble ECE	0.0912
Representative model	Seed 44
Raw ECE	0.0792
Calibrated ECE	0.0310
ECE reduction	≈60.9%
Cochran's Q p-value	0.877718
Bootstrap ensemble macro-F1	0.8355
Bootstrap 95% CI	0.7614–0.8857

The reliability analysis shown in **Figure 8** also shows the impact of temperature scaling, with ECE dropping from 0.0792 to 0.0310 and moving closer to ideal calibration on the confidence–accuracy curve.

**Figure 8:**
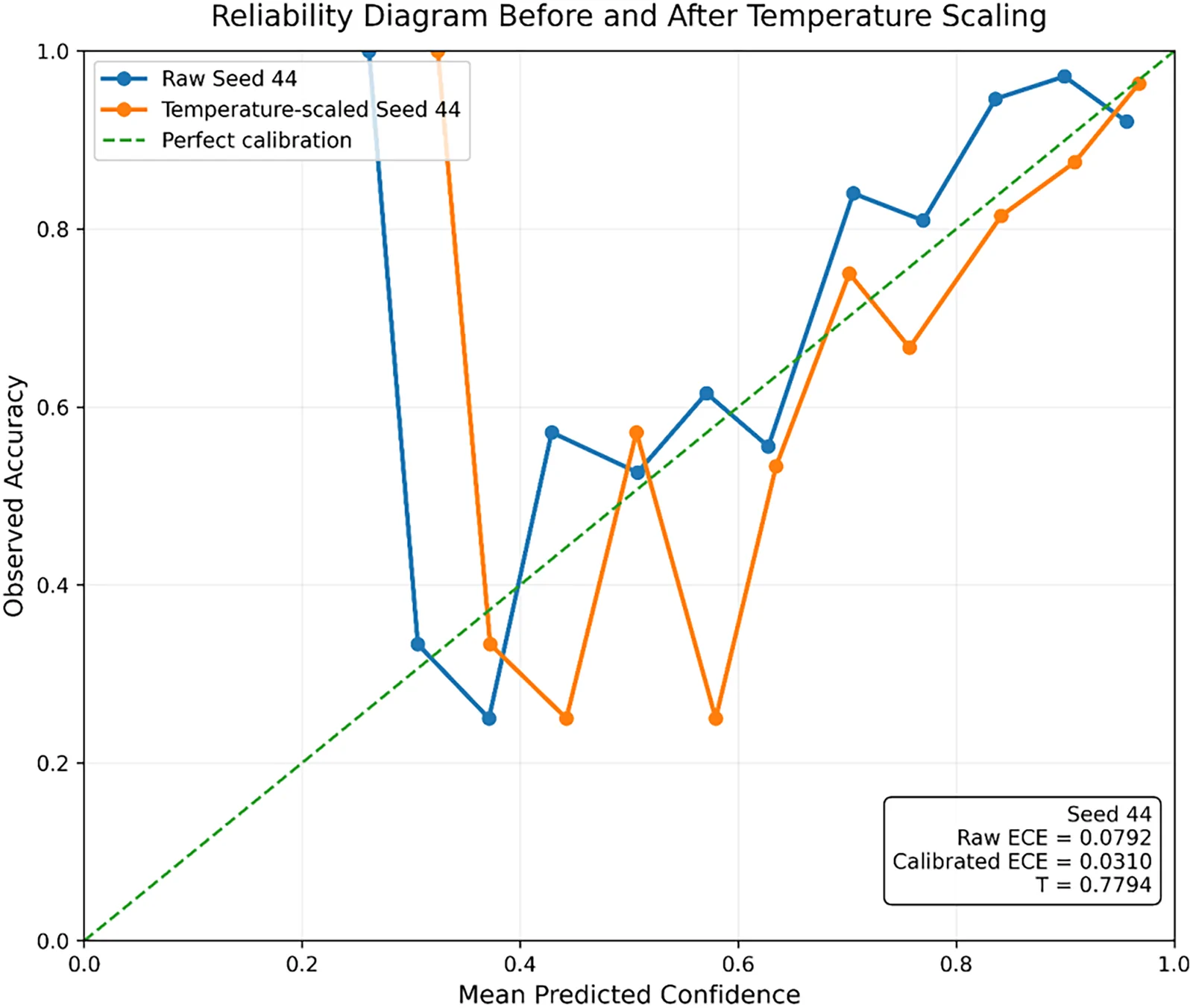
Reliability analysis of the representative AgroX-Swin model before and after temperature scaling.

The reliability curve for the representative seed-44 is computed from the locked held-out test set, and the diagonal corresponds to perfect calibration. Temperature scaling improved model calibration, reducing the Expected Calibration Error (ECE) from 0.0792 to 0.0310 at an optimized temperature of T = 0.7794. The calibrated confidence estimates show improved overall agreement with observed accuracy, supporting more reliable interpretation of the model’s predictive confidence.

### 4.4 Quantitative XAI fidelity, transformation stability, and model randomization

The highlighted areas are quantitatively assessed to check if they are meaningful to the model predictions, called Grad-CAM++. The mean confidence drop on perturbing highly attributed regions is 0.2305, and 84.0% of the cases analyzed do show a positive confidence drop. This suggests that the focus of Grad-CAM++ around these regions typically carries information that aids in predicting the class. A controlled fidelity analysis is conducted on the predefined class-balanced subset of 42 held-out images using Grad-CAM++, SHAP, LIME, all three pairwise fusions, and the equal-weight three-way Multi-XAI fusion. At the predefined 20% perturbation level, the mean confidence drops for high-attribution regions are:

As shown in **Table 8**, perturbation-based fidelity varies across the individual and fused XAI methods at the predefined 20% perturbation level. Grad-CAM++ + LIME produces the largest mean confidence drop, followed closely by the three-way Multi-XAI fusion. These results indicate complementary prediction-relevant attribution behavior rather than universal superiority of any single XAI or fusion strategy.

**Table 8:** Perturbation-based fidelity of individual and fused XAI methods at the 20% perturbation level.

Explanation method	Mean confidence drop
Grad-CAM++	0.2414
SHAP	0.0973
LIME	0.2046
Grad-CAM++ + SHAP	0.2317
Grad-CAM++ + LIME	0.2540
SHAP + LIME	0.2332
Grad-CAM++ + SHAP + LIME	0.2507

Grad-CAM++ + LIME (0.2540) shows the largest numerical decrease in confidence, closely followed by Multi-XAI fusion (0.2507). These differences are merely descriptive and should not be considered as evidence of any general superiority of any fusion.

The mean confidences at 20% perturbation for the equal-weight three-way Multi-XAI fusion are 0.2507, 0.2256, and 0.0771 for high-, matched random regions, and low-attribution regions, respectively. The high-attribution (HA) versus low-attribution (LA) contrast is statistically significant after Holm correction, and the high-attribution (HA) versus random-region (RR) contrast is not statistically significant post-Holm. Thus, highly attributed regions have a greater impact than low-attributed regions, but not superiority over matched random-region perturbation, as shown by the analysis. Transformation-based stability is evaluated under brightness adjustment, Gaussian noise, +4-pixel translation, and horizontal flipping. Across all 168 transformed cases (42 images × four transformations), the originally predicted class is preserved in 100% of cases under these predefined mild transformations.

The results summarized in **Table 9** reveal distinct stability characteristics among the individual and fused XAI methods on the class-balanced held-out subset. Grad-CAM++ records the highest transformation stability, while SHAP + LIME achieves the highest attribution-burden stability. The observed differences highlight complementary strengths of the explanation methods rather than the consistent superiority of any single XAI configuration.

**Table 9:** Comparative transformation stability and attribution-burden stability of individual and fused XAI methods on the class-balanced held-out subset.

Explanation method	Mean transformation-stability spearman	Attribution-burden stability
Grad-CAM++	**0.8371**	0.7485
SHAP	0.7010	0.7714
LIME	0.3612	0.6417
Grad-CAM++ + SHAP	0.8173	0.7489
Grad-CAM++ + LIME	0.6407	0.7543
SHAP + LIME	0.5439	**0.7954**
Three-way Multi-XAI	0.6963	0.7855

Bold values indicate values highlighted for emphasis within the corresponding table.

Grad-CAM++ achieved the highest transformation-stability Spearman correlation (0.8371), whereas SHAP + LIME achieved the highest attribution-burden stability (0.7954). The three-way Multi-XAI fusion showed intermediate transformation stability (0.6963) and comparatively high attribution-burden stability (0.7855), indicating that different explanation configurations exhibit different strengths rather than a uniformly superior fusion strategy.

The Representative Grad-CAM++ visualizations are provided in **Figure 9**, with the spatial regions that contribute to the model predictions presented for each species–health class. The activation maps and associated overlays suggest that the model focuses more on regions of the leaves that contain discriminative visual patterns.

**Figure 9:**
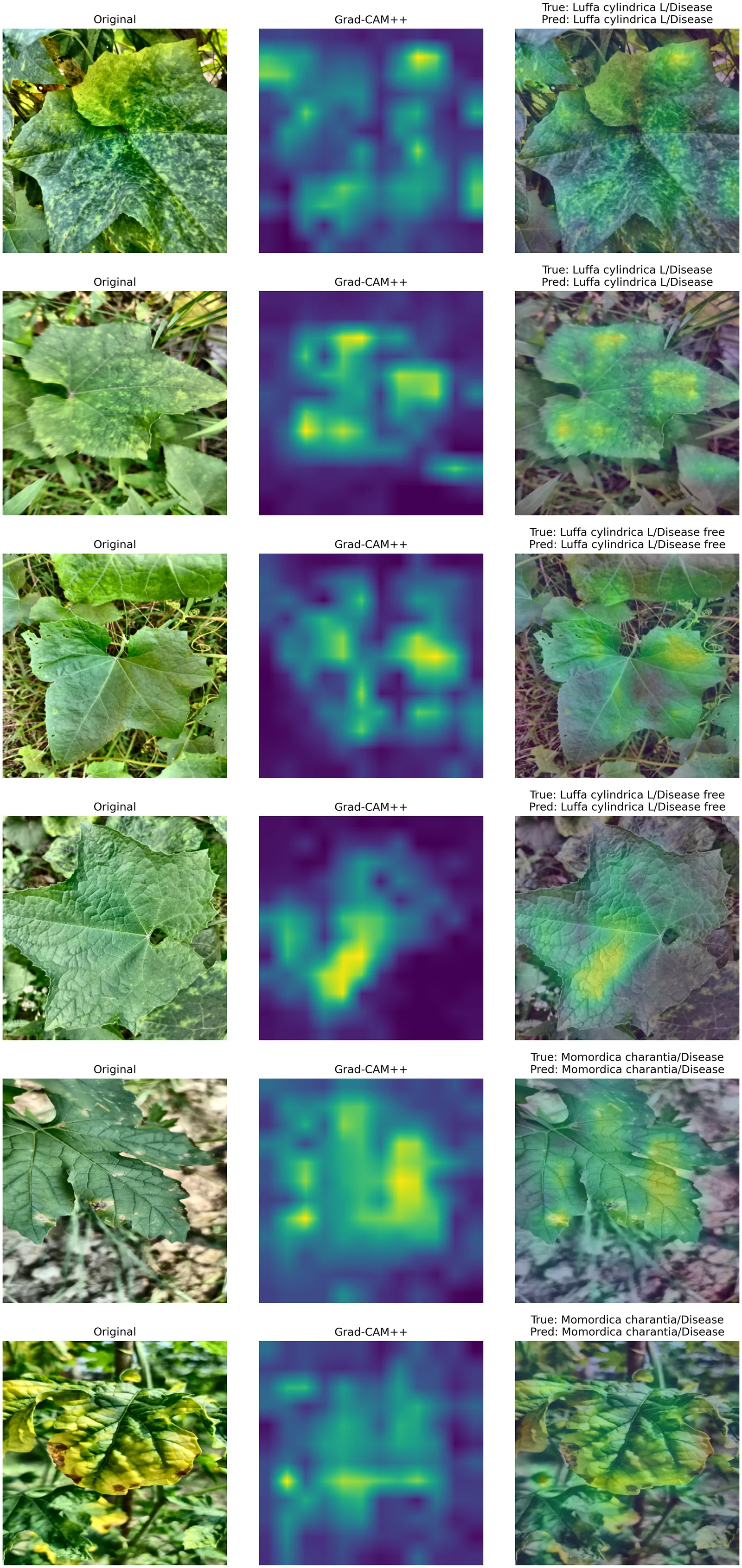
Representative Grad-CAM++ explanations generated by the final AgroX-Swin model.

The images in each row show the input image, input image Grad-CAM++, and the heatmap overlaid on the input image. The visualizations show the spatial zones that feed into the predictions made by the model for representative species–health classes.

Here, explanations using brightness variation, Gaussian noise, rotation, and translation are further assessed using transformation-based stability analysis. Across all transformations, the prediction-preservation rate is 0.9750, and the mean Pearson and Spearman correlations are 0.9743 and 0.9610, respectively. The mean thresholded IoU is 0.8612, and the mean absolute CAM difference is 0.0273.

The explanation maps remain particularly stable under brightness and Gaussian-noise perturbations, while geometric transformations produce relatively larger spatial changes. However, overall attribution stability remains high.

The Grad-CAM++ model-randomization sanity test is performed on 30 held-out images using three independent randomization conditions. Reinitializing the classification head yields a mean Pearson correlation of 0.1071, mean top-region IoU of 0.2236, and mean absolute CAM difference of 0.2219 relative to the trained model. Randomizing the final Swin feature stage yields corresponding values of 0.3794, 0.2970, and 0.2011, while randomizing the final two feature stages yields 0.1317, 0.1962, and 0.2380, respectively.

The substantial changes in the attribution maps following parameter randomization indicate that Grad-CAM++ depends on learned model parameters. However, the three conditions do not form a monotonic cumulative sequence and should not be interpreted as demonstrating progressively increasing degradation with network depth.

Representative Grad-CAM++ (GC) maps and overlays, showing the spatial areas that make up the model predictions of the representative species–health classes, are displayed in **Figure 9**. The quantitative results on fidelity and stability are shown in **Table 10**, and quantitative results on stability and model-randomization are provided in the **Supplementary Material** in a transformation-wise manner.

**Table 10:** Grad-CAM++ quantitative validation.

Evaluation	Result
Mean confidence drop	0.2305
Positive confidence-drop rate	84.0%
Prediction preservation under transformations	0.9750
Mean Pearson correlation	0.9743
Mean Spearman correlation	0.9610
Mean thresholded IoU	0.8612
Mean absolute CAM difference	0.0273

### 4.5 Cross-explainer agreement, fusion sensitivity, and computational trade-offs

Cross-explainer analysis on the 42-image subset demonstrates limited spatial correspondence among Grad-CAM++, SHAP, and LIME. The mean pairwise Spearman spatial agreement is approximately 0.270, while the mean top-25% attribution IoU is approximately 0.246. Image-level attribution-burden ranks show a higher but still limited mean pairwise Spearman agreement of approximately 0.483.

These results indicate that the three explainers frequently emphasize different regions of the same image. Such disagreement should not automatically be interpreted as explanation failure because Grad-CAM++, SHAP, and LIME rely on different explanation mechanisms. Instead, the results support their use as complementary explanatory views rather than interchangeable explanations.

Further to assess whether the three-way fusion depends strongly on equal weighting, the equal-weight map is compared with three predefined unequal weighting schemes. Across these comparisons, mean Pearson correlation is 0.971, mean Spearman correlation is 0.974, and mean top-25% IoU is 0.794. The high correspondence indicates that modest changes in prespecified explainer weights do not substantially alter the overall fused attribution pattern. Equal weighting is therefore retained as a transparent, non-optimized fusion rule rather than as an empirically optimal weighting scheme.

The comparative evaluation in **Table 11** highlights the trade-offs between explanation performance and computational cost across the individual and fused XAI methods. Grad-CAM++ offers the lowest computational cost with strong transformation stability, whereas different fusion strategies provide advantages in fidelity or attribution-burden stability. Overall, the results indicate that no single XAI configuration performs best across all evaluation criteria.

**Table 11:** XAI performance and computational trade-offs.

Method	Fidelity at 20%	Transformation stability	Burden stability	Runtime (s/image)
Grad-CAM++	0.2414	0.8371	0.7485	0.0834
SHAP	0.0973	0.7010	0.7714	5.1638
LIME	0.2046	0.3612	0.6417	13.1000
GC++ + SHAP	0.2317	0.8173	0.7489	5.2513
GC++ + LIME	**0.2540**	0.6407	0.7543	13.1874
SHAP + LIME	0.2332	0.5439	**0.7954**	18.2678
Three-way Multi-XAI	0.2507	0.6963	0.7855	18.3512

Bold values indicate values highlighted for emphasis within the corresponding table.

No single explanation configuration is uniformly superior across all criteria. Grad-CAM++ provides the highest transformation stability and lowest computational cost, Grad-CAM++ + LIME gives the largest numerical fidelity value at the predefined 20% perturbation level, and SHAP + LIME gives the highest attribution-burden stability. The three-way fusion provides comparatively strong performance across several criteria but incurs the highest computational cost and is not interpreted as universally superior.

Representative explanations are provided for the three XAI methods and their equally averaged fusion in **Figure 10**. Grad-CAM++ offers coarse spatial localization of important regions, while SHAP and LIME offer complementary attribution patterns at various spatial granularities. These different explanations are merged into one visualization in the fused maps. Importantly, these highlighted areas are not lesion segmentations, but rather areas that help predict the model.

**Figure 10:**
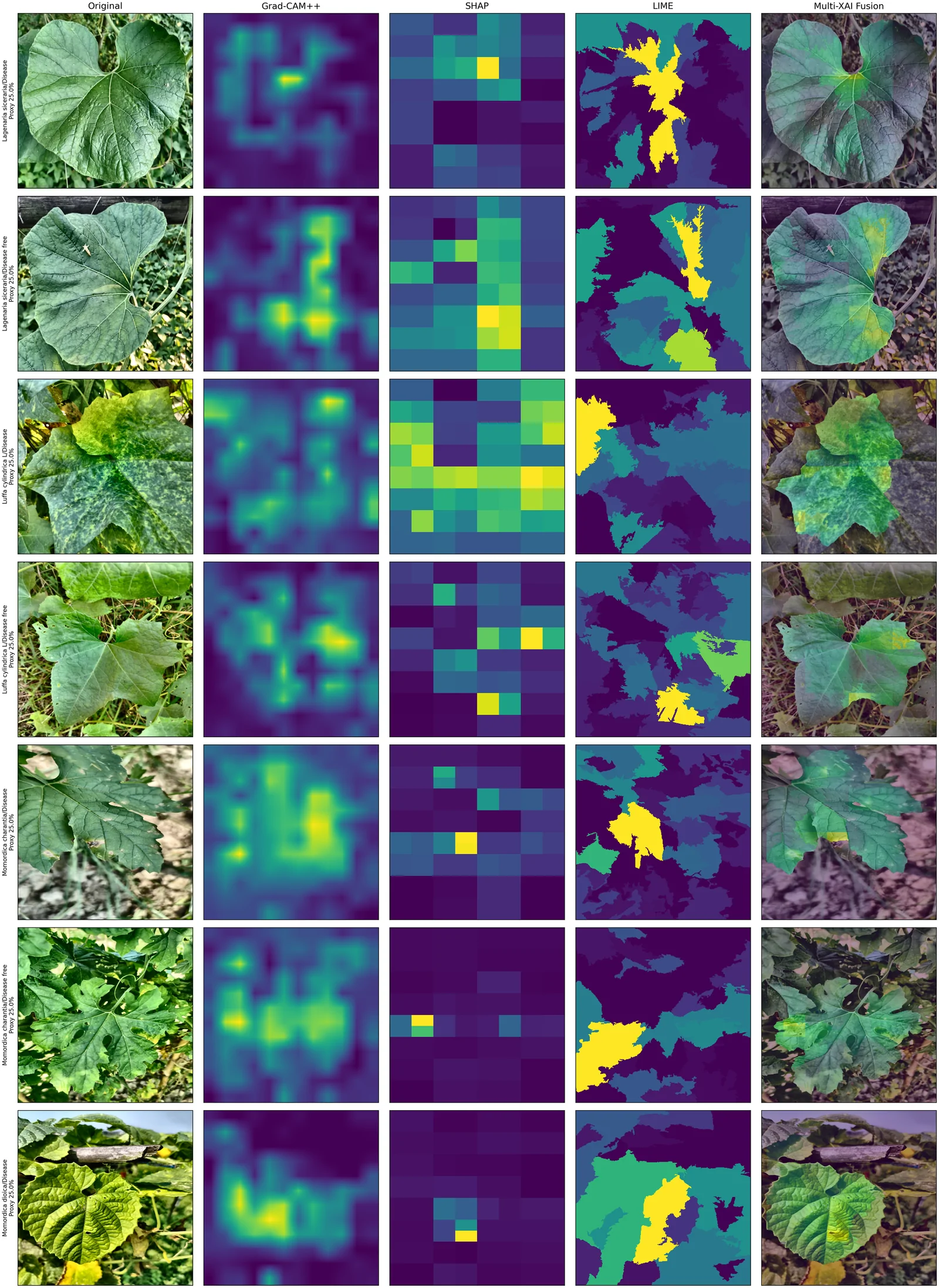
Representative Multi-XAI explanations for the final AgroX-Swin model.

Original RGB images are displayed along with Grad-CAM++, SHAP, LIME, and equal-weight Multi-XAI fusion maps for representative Disease and Disease-free samples for various plant species. Explanation methods offer complementary spatial attribution patterns, and the fused maps summarize the combined evidence. Highlighted areas show areas of the image that are relevant for the prediction and should not be interpreted as pixel-level disease lesions or quantitative estimates of disease severity.

As the agreement is only partial, it confirms that the three approaches explain different patterns of attribution as might be predicted, given that they use different explanation mechanisms. The normalized maps are thus fused with an equal-weight Multi-XAI fusion, instead of optimizing the weights of the individual explainers afterward.

The agreement and attribution analyses of the complementary XAI methods are summarized in **Table 12**, showing moderate pairwise agreement but strong threshold-rank stability and burden-measure consensus.

**Table 12:** Multi-XAI agreement and attribution analysis.

Analysis	Result
Grad-CAM++/SHAP correlation	0.3048
Grad-CAM++/LIME correlation	0.2611
SHAP/LIME correlation	0.2552
Mean pairwise XAI agreement	0.2737
Threshold-rank stability	0.7969
Burden-measure consensus	0.9418
Diseased–healthy consensus difference	−1.53 percentile points
Bootstrap 95% CI of difference	−30.36 to 29.34

Further analysis shows that there is a lot of variation in the estimates for affected regions depending on the threshold. The MAD-based approach yields an average proxy of 16.90 ± 6.54% while the adaptive Otsu thresholding approach yields 40.93 ± 16.05%. They both have negative Pearson (−0.7325) and Spearman (−0.7626) correlations, which show that there is a significant difference of opinion between thresholding methods.

The threshold-independent analysis reveals a threshold rank stability of 0.7969 and a high burden-measure consensus of 0.9418, suggesting that relative, rather than absolute threshold-derived percentages of attribution, are more stable. However, the difference between the Disease–Disease-free consensus is just −1.53 percentile points, ranging from −30.36 to 29.34 on the wide 95% bootstrap confidence interval. Thus, it is not appropriate to use the percentages if they can be derived from XAI as a measure of biological disease severity.

(a) Distribution of leaf area above a set threshold for each of the normalized Multi-XAI attribution values over a representative held-out sample. Each colored curve represents an individual test image and shows the percentage of leaf area exceeding normalized Multi-XAI attribution thresholds from 0.1 to 0.9. The progressive reduction in attributed area at higher thresholds demonstrates the sensitivity of the estimated attribution burden to threshold selection (b) Spearman correlation among the complementary measures of attribution-burden independent of the threshold. The analyses illustrate the threshold-likely nature of absolute spatial attribution estimates, and reveal greater consistency between the relative measures of attribution burden.

The final Multi-XAI maps are therefore interpreted as qualitative spatial explanations and relative attribution-burden evidence rather than quantitative lesion localization or valid estimates of disease severity. This differentiation is of great significance as prediction attribution can be used to define areas that contribute to the model’s decision-making process, but not necessarily the areas of diseased tissue.

**Figure 10** presents representative Original RGB images together with the corresponding Grad-CAM++, SHAP, LIME, and fused Multi-XAI maps, while **Figure 11** summarizes threshold sensitivity and attribution-burden consensus.

**Figure 11:**
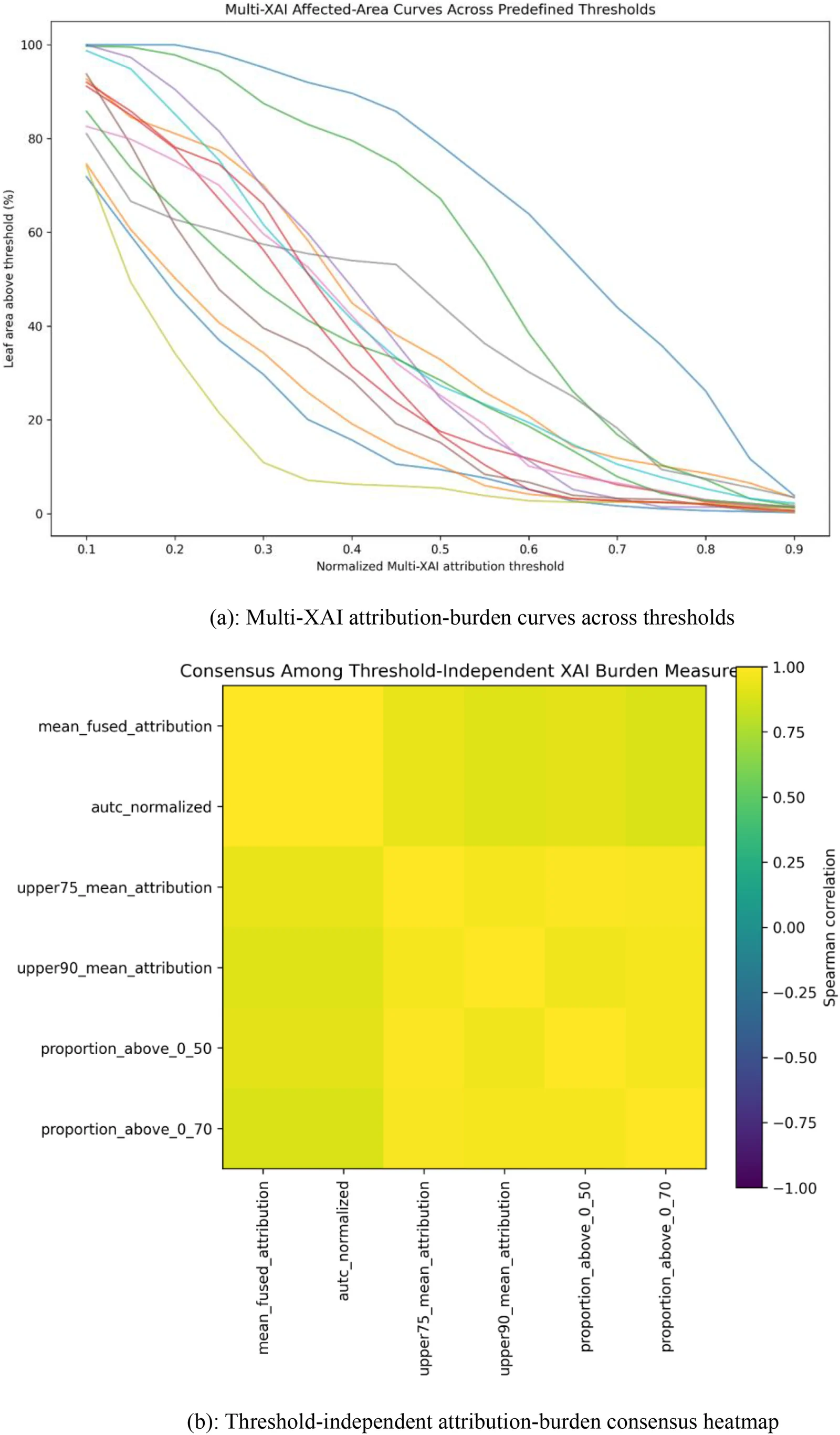
**(a)** Multi-XAI attribution-burden curves across thresholds. **(b)** Threshold-independent attribution-burden consensus heatmap. Threshold-sensitivity and attribution-consensus analysis of fused Multi-XAI explanations.

### 4.6 Attribution-burden analysis and biological interpretation

Attribution-burden analysis is employed to describe the relative area of the predicted leaf region attributed to the prediction. Historical threshold-based analysis shows that absolute attribution percentages are sensitive to the threshold. In fact, absolute threshold-based approaches (MAD-based and adaptive Otsu) yield significantly different spatial percentage values, so absolute threshold-based values may not reflect the extent of physical disease.

This is because threshold-independent analysis methods summarize the relative attribution burden. However, they are still model-centric explanation summary quantities. The threshold-rank stability and burden-measure consensus are 0.7969 and 0.9418, respectively, and they reflect agreement between different attribution-burden summaries, not spatial agreement among the three models; these numbers should not be confused with the spatial agreement between Grad-CAM++, SHAP, and LIME.

The Disease vs Disease-free comparison of attribution burden also doesn’t support a 1:1 relationship between attribution area and the extent of biological disease. The XAI outputs are thus regarded not as lesion segmentation, but as prediction-relevant visual attributions and relative attribution-burden measures. Without expert-annotated lesion masks or validated disease-severity ground truth, the outputs of the XAI are not regarded as a disease-severity estimate, but rather as a measure of the relative value attributed to the visual attributes.

### 4.7 Overall discussion

Overall, the experiments yield several disclaimed conclusions. Original RGB with Swin-T is the best option in terms of validation performance of the preprocessing/architecture options studied in the controlled development protocol. Second, the five runs in the five runs sections do not vary much, while the five models in the ensemble only give a small improvement in numbers over the individual models. Third, species identity is recognized much more accurately than binary health status, with the latter being a more challenging aspect of the joint task and thus not interpreted as performance in detecting diseases. Fourth, uncertainty remains for held-out macro-F1 scores due to a small test sample (class-imbalanced), and validation-fitted temperature scaling significantly improves the calibration of the representative classifier. Finally, the XAI experiments illustrate complementary properties among Grad-CAM++, SHAP, and LIME, showing that the methods trade off differently across perturbation fidelity, stability in transform space, stability in attribution-burden space, and computational cost.

The results of the present work thus demonstrate that AgroXAI would be effective as a controlled framework within a dataset to assess both classification and calibration, uncertainty, and complementary XAI behavior. They do not demonstrate robustness across datasets, field generalization, localization of a biological lesion, quantitative disease severity, or superior overall performance of Multi-XAI fusion. These attributes require external, independent datasets and expert-annotated lesion information.

## 5 Limitations

The following are the two major limitations of the present study: First, the group-disjoint experimental design allows for a solid held-out test of the system’s efficacy, but independent held-out data sets and field-acquired images would strengthen evidence of the system’s generalizability across acquisition and environmental conditions. Second, expert-annotated lesion masks and disease-severity ground truth are not available, so the Multi-XAI maps are used as qualitative spatial explanations and relative attribution-burden evidence, rather than quantitative estimates of the lesion or disease severity. For future research, external validation in the field will be performed, as well as expert-annotated localization analysis.

## 6 Conclusion

This study introduces the AgroXAI framework, an integrated approach combining Deep Learning and Multi-XAI for multi-species plant disease classification in an experimental protocol with no leakage. The framework brings together data-integrity auditing, group-disjoint partitioning, validation-based preprocessing/architecture selection, training-stability analysis using multiple seeds, held-out classification, predictive calibration, statistical uncertainty analysis, and complementary explainability evaluation under a single roof. The AgroX-Swin classifier proves successful for joint species–health class discrimination, and multi-level evaluation indicates that species recognition is more difficult than discrimination between disease and disease-free. Discriminability analysis also shows that the explainability methods, individually and in combination, offer complementary views on the visual evidence pertinent to the prediction: explainability methods have different “perturbation fidelity,” “transformation stability,” “attribution-burden stability,” “parameter sensitivity,” and “computational cost” trade-offs. The results show that no single explanation or fusion method is consistently best across all evaluation criteria, highlighting the need to look beyond qualitative XAI visualizations and include quantitative evaluation. Importantly, the resulting attribution maps and attribution-burden measures explain what is going on in the model, not lesion localization or a quantitative estimate of disease severity. In general, AgroXAI offers a structured approach to concurrently explore predictive performance, calibration, uncertainty, and complementary explanation behavior attributes; however, the present results have been obtained with a within-dataset held-out evaluation, and future studies should consider independently acquired datasets from field acquisitions, varying acquisition settings, wider computational parameters, and expert-annotated lesion information to evaluate external generalization and biologically valid explanations.

## 7 Future directions

Future studies will aim to develop AgroXAI further to allow for independent testing on larger and more diverse multi-species datasets from different environmental, geographical, and imaging conditions in the field. Adding expert-annotated lesion masks and disease-severity scores will allow for direct validation of localization by the XAI and explore the relationship between localization by the XAI and the extent of the disease. Lightweight transformer architectures, Knowledge Distillation, pruning, and quantization can be explored further to facilitate deployment of transformers on mobile and edge devices. Furthermore, domain adaptation and continual learning can be explored to enhance the adaptability to unseen environments, novel diseases, and new plant species, enabling more trustworthy and explainable plant health monitoring solutions to be put into practice in precision agriculture for real-world applications.

## Data Availability

Publicly available datasets were analyzed in this study. This data can be found here: https://data.mendeley.com/datasets/v46jkbbzv3/2.
