# Supplementary material for: AgroXAI: a multi-XAI deep learning system for explainable multi-species plant disease classification
Source: Front Plant Sci. 2026 Oct 14;17:1965848. doi: 10.3389/fpls.2026.1965848 (PMC13713886; doi:10.3389/fpls.2026.1965848)
Supplement: Supplementary file 1 [file SupplementaryFile1.pdf]

Supplementary Material

AgroXAI: a multi-XAI deep learning system for explainable multi-species plant disease classification

Authors: Sathish Kumar K. and G. N. Balaji

Journal: Frontiers in Plant Science

This supplementary document consolidates the final experimental evidence, reproducibility information, leakage-control checks, validation-only model-selection results, locked held-out test results, calibration analysis, explainability validation, and statistical analyses produced by the executed AgroXAI revision pipeline.

Important scope: This document reports supporting evidence only. Attribution maps are prediction-relevant explanations and are not expert-validated lesion segmentations or quantitative agronomic disease-severity measurements.

Contents

Section	Content
S1	Supplementary scope and evidence policy
S2	Dataset composition and source-group integrity audit
S3	Leakage-aware group-disjoint partitioning
S4	Preprocessing representations and validation-only screening
S5	Architecture screening
S6	Advanced enhancement screening and configuration lock
S7	Final five-seed training and locked held-out evaluation
S8	Multi-level evaluation and uncertainty
S9	Calibration and temperature scaling
S10	Grad-CAM++ quantitative validation
S11	Transformation stability and model-randomization checks
S12	Statistical comparison across seeded models
S13	Multi-XAI interpretation and attribution-burden limitation
S14	Reproducibility checklist and supplementary captions

The material is intentionally condensed from the full executed notebook archive so that the supplementary file remains readable and publication-oriented.

S1. Supplementary scope and evidence policy

The AgroXAI revision workflow separates development evidence from the final held-out test. Preprocessing, architecture, enhancement, checkpoint selection, and calibration development are performed without using the held-out test for model selection.

The final configuration entering the locked test stage is: **Original RGB -> Swin Transformer-Tiny -> 384 x 384 -> E0 baseline -> no test-time augmentation**. Independent training seeds are 11, 22, 33, 44, and 55.

The held-out test contains 423 images and is revealed only after the five final checkpoints have been completed. No post-test return to preprocessing, architecture, enhancement, or hyperparameter selection is permitted.

Evidence hierarchy

Evidence class	Use in the pipeline
Training split	Parameter optimization and stochastic augmentation
Validation split	Preprocessing, architecture, enhancement, checkpoint and calibration development
Held-out test	One-time final evaluation after configuration lock
XAI subset	Post-hoc explainability analyses on the locked final model family

S2. Dataset composition

The cleaned dataset contains **2,815 leaf images** organized into **14 joint species-health classes**, representing seven species under Disease and Disease-free states.

Class	Images
Lagenaria siceraria / Disease	391
Lagenaria siceraria / Disease-free	146
Luffa cylindrica L / Disease	141
Luffa cylindrica L / Disease-free	45
Momordica charantia / Disease	275
Momordica charantia / Disease-free	25
Momordica dioica / Disease	349
Momordica dioica / Disease-free	189
Phaseolus vulgaris L / Disease	311
Phaseolus vulgaris L / Disease-free	206
Solanum melongena L / Disease	204
Solanum melongena L / Disease-free	38
Vigna unguiculata subsp. sesquipedalis / Disease	316
Vigna unguiculata subsp. sesquipedalis / Disease-free	179

S2. Source-group integrity and duplicate audit

Source-group reconstruction retained a group only when supported by exact SHA-256 duplicate evidence, perceptual near-duplicate evidence, or manually confirmed same-source review. Filename similarity alone was not accepted as source evidence.

Audit item	Final result
Total images	2,815
Evidence-supported groups	2,815
Singleton images	2,815
Remaining exact SHA-256 duplicate groups	0
Perceptual near-duplicate groups	0
Manually confirmed same-source-group images	0
Filename heuristic used as grouping evidence	False
Classes with fewer than three independent groups	0
Final audit verdict	PASS

The audit therefore produced an evidence-supported manifest in which all retained images were independent singleton groups for the subsequent split stage.

S3. Leakage-aware group-disjoint partitioning

The fixed split targets approximately 70:15:15 for training, validation, and held-out test partitions. The final counts are **1,970 training**, **422 validation**, and **423 held-out test** images.

Partition	Images	Primary role
Training	1,970	Model parameter optimization; stochastic augmentation after splitting
Validation	422	Development-stage selection and calibration fitting
Held-out test	423	Final locked evaluation only

Partition integrity is checked using image identifiers, file paths, and evidence/source-group identifiers. The development protocol is designed to prevent the held-out test from influencing preprocessing, architecture, enhancement, checkpoint, or hyperparameter decisions.

S3. Partition and evaluation safeguards

Safeguard	Status
Group-aware splitting performed after evidence-supported source-group reconstruction	Applied
All 14 joint classes represented across the fixed experimental partitions	Applied
Training-only stochastic augmentation	Applied
Validation/test augmentation for training	Not used
Held-out test used for preprocessing selection	No
Held-out test used for architecture selection	No
Held-out test used for enhancement selection	No
Held-out test used for checkpoint selection	No
Post-test tuning permitted	No

These safeguards support the manuscript's leakage-aware evaluation design and preserve the held-out test as a final assessment set.

S4. Preprocessing representations

Three matched input representations were screened on the fixed development partitions: Original RGB, Hard U2Net, and Soft U2Net. Foreground extraction was not assumed a priori to improve classification.

Representation	Description
Original RGB	Complete leaf image and surrounding context retained.
Hard U2Net	U2Net-derived saliency converted to a binary foreground mask.
Soft U2Net	Saliency used as a continuous weighting function that attenuates rather than fully removes context.

Selection was based on validation macro-F1 only; the held-out test remained unevaluated during this stage.

S4. Validation-only preprocessing screening

Variant	Best epoch	Validation macro-F1	Validation loss
Original RGB	17	0.8215	0.9457
Soft U2Net	12	0.7983	0.9616
Hard U2Net	17	0.7663	1.0795

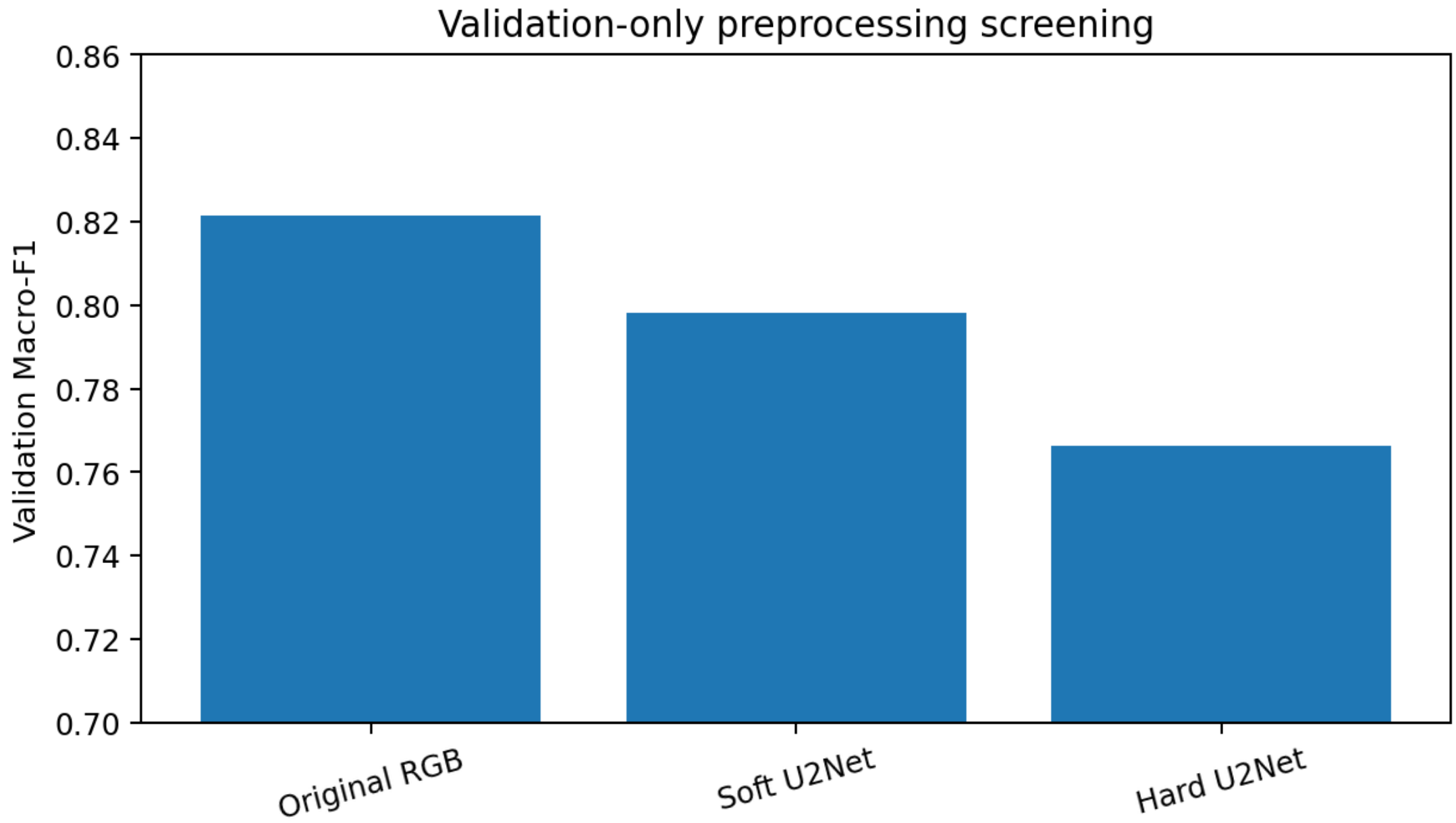

Supplementary Figure S1. Validation-only preprocessing screening. Original RGB achieved the highest validation macro-F1 and was selected for architecture screening.

S5. Architecture screening

Candidate architectures were compared using the selected Original RGB representation. Selection again used validation macro-F1 only.

Architecture	Best epoch	Validation macro-F1	Validation loss
Swin-T	12	0.8394	0.9279
ConvNeXt-Tiny	17	0.8187	1.0162
EfficientNetV2-S + CBAM	11	0.7983	0.9270

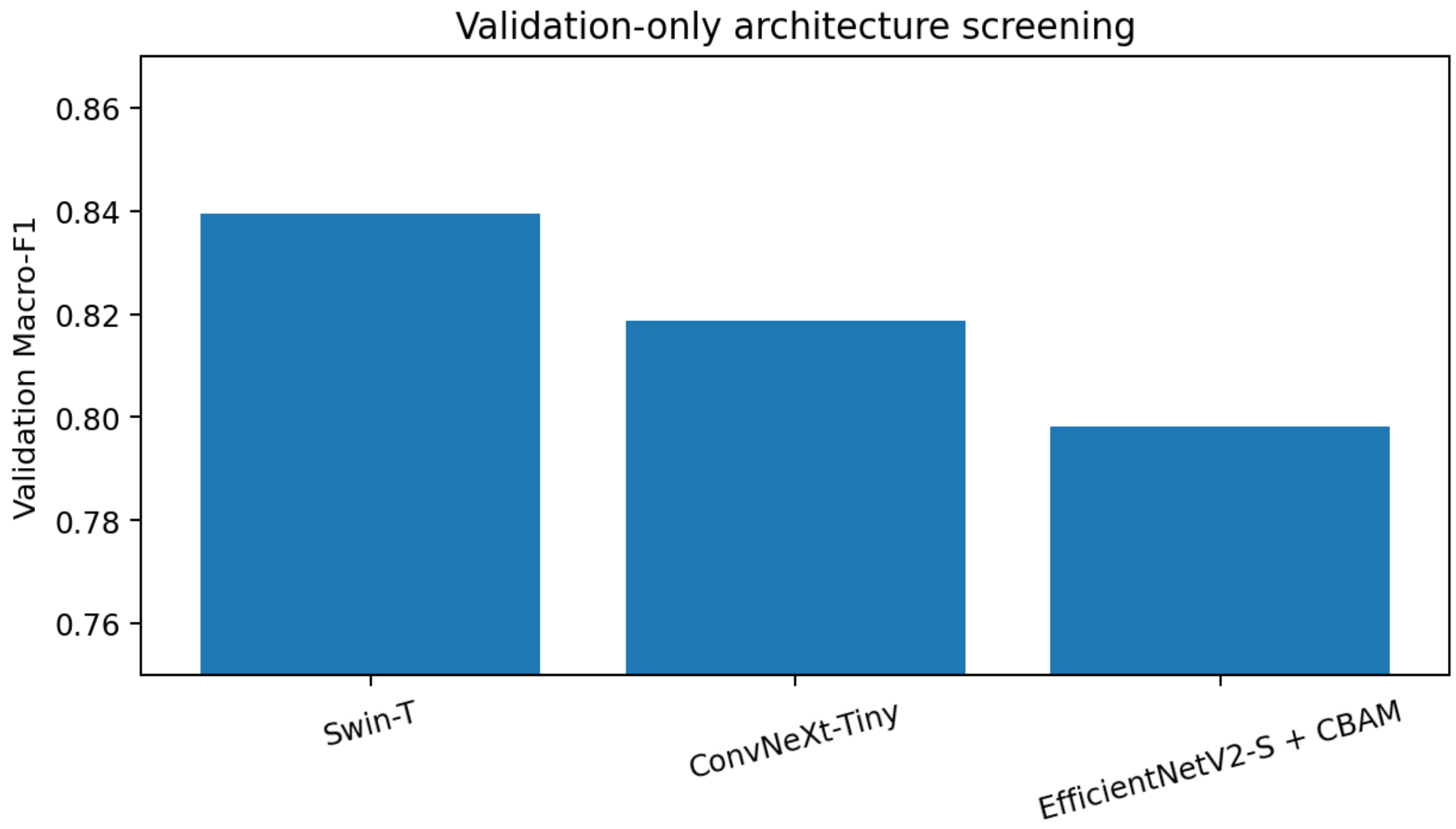

Supplementary Figure S2. Validation-only architecture screening. Swin-T was selected.

S6. Advanced enhancement screening and configuration lock

Item	Result
Base architecture	Swin-T
Baseline validation macro-F1	0.8473
Selected enhancement experiment	E0 baseline
Enhanced validation macro-F1	0.8473
Absolute macro-F1 improvement	+0.0000
Validation TTA macro-F1	0.8449
TTA selected	False
Final selected validation macro-F1	0.8473
Held-out test evaluated at this stage	No
Final verdict	PASS

The baseline configuration was retained because the screened enhancement alternatives did not improve the validation criterion. Test-time augmentation was also not selected.

S7. Final five-seed training and locked held-out test

The final model family was trained independently with seeds **11, 22, 33, 44, and 55**. The configuration was locked before test access. The held-out test contained 423 images.

Final aggregate measure	Result
Mean held-out test accuracy	0.8719
Mean held-out test macro-F1	0.8300
SD of test macro-F1	0.0122
95% CI across five runs for test macro-F1	[0.8148, 0.8451]
Five-model probability ensemble accuracy	0.8794
Five-model probability ensemble macro-F1	0.8355
Test used for model selection	No
Final verdict	PASS

S7. Multi-level held-out evaluation

Evaluation level	Result
Primary 14-class ensemble accuracy	0.8794
Primary 14-class ensemble macro-F1	0.8355
Deterministically aggregated species accuracy	0.9929
Deterministically aggregated binary health accuracy	0.8842
Diseased-class F1	0.9160

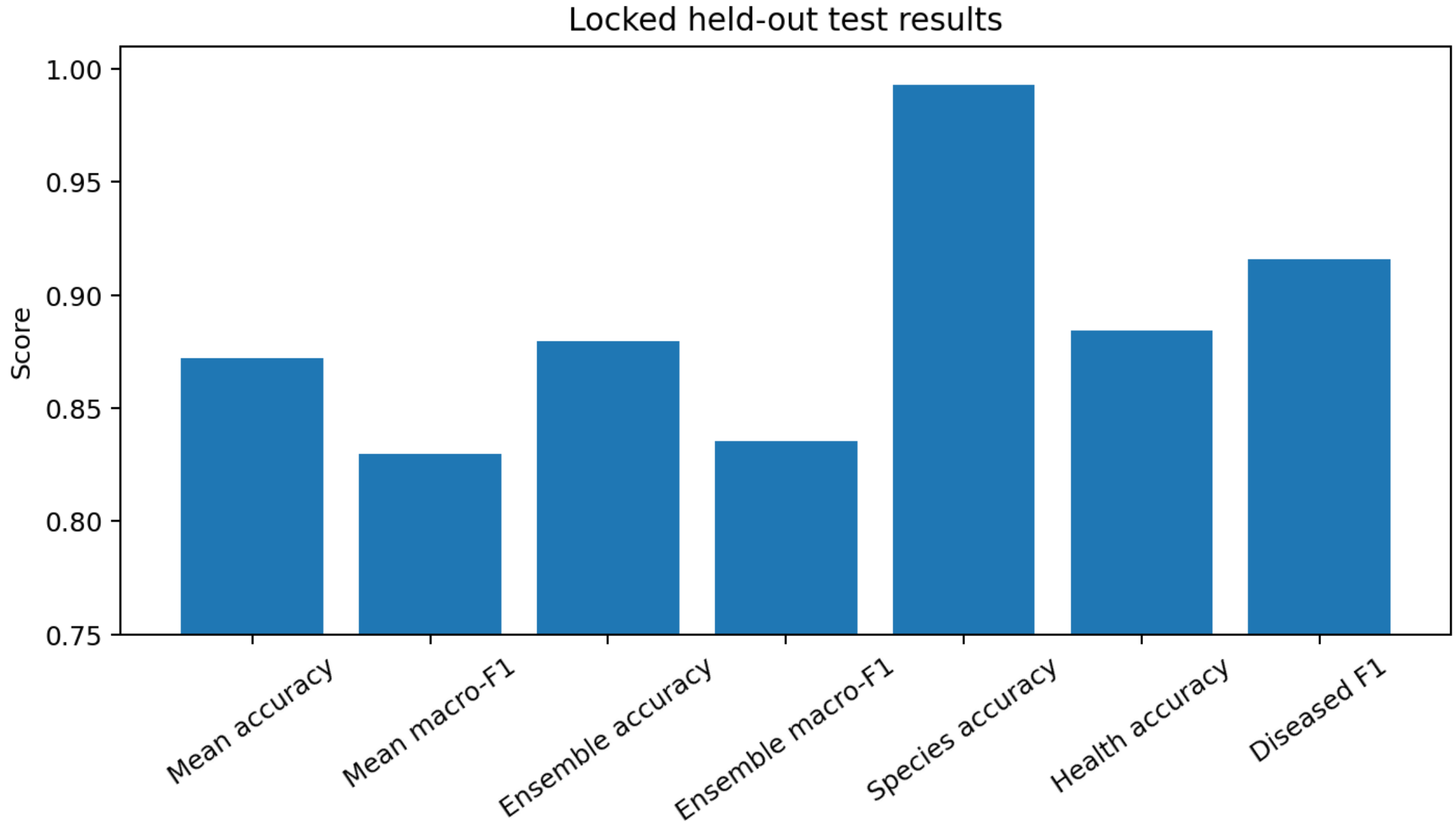

Supplementary Figure S3. Locked held-out performance of the final model family and five-model probability ensemble.

S8. Statistical uncertainty of the final ensemble

Bootstrap confidence intervals were computed using 5,000 resamples of the held-out test predictions.

Metric	Estimate	Bootstrap mean	95% bootstrap CI
Ensemble accuracy	0.8794	0.8799	[0.8487, 0.9102]
Ensemble macro-F1	0.8355	0.8271	[0.7614, 0.8857]

The macro-F1 interval is wider than the accuracy interval because macro-F1 gives equal importance to each of the 14 classes, including classes with relatively small support.

S9. Predictive calibration

The representative model was selected from the already-finalized five seeds using validation performance only. The representative seed was **44**.

Calibration quantity	Raw	Temperature-scaled
Expected Calibration Error (ECE)	0.0792	0.0310
Negative log-likelihood	0.4765	0.4440
Multiclass Brier score	0.1958	0.1895

Temperature scaling was fitted on the validation split only. The fitted temperature was **T = 0.7794**; no test labels were used to fit the temperature.

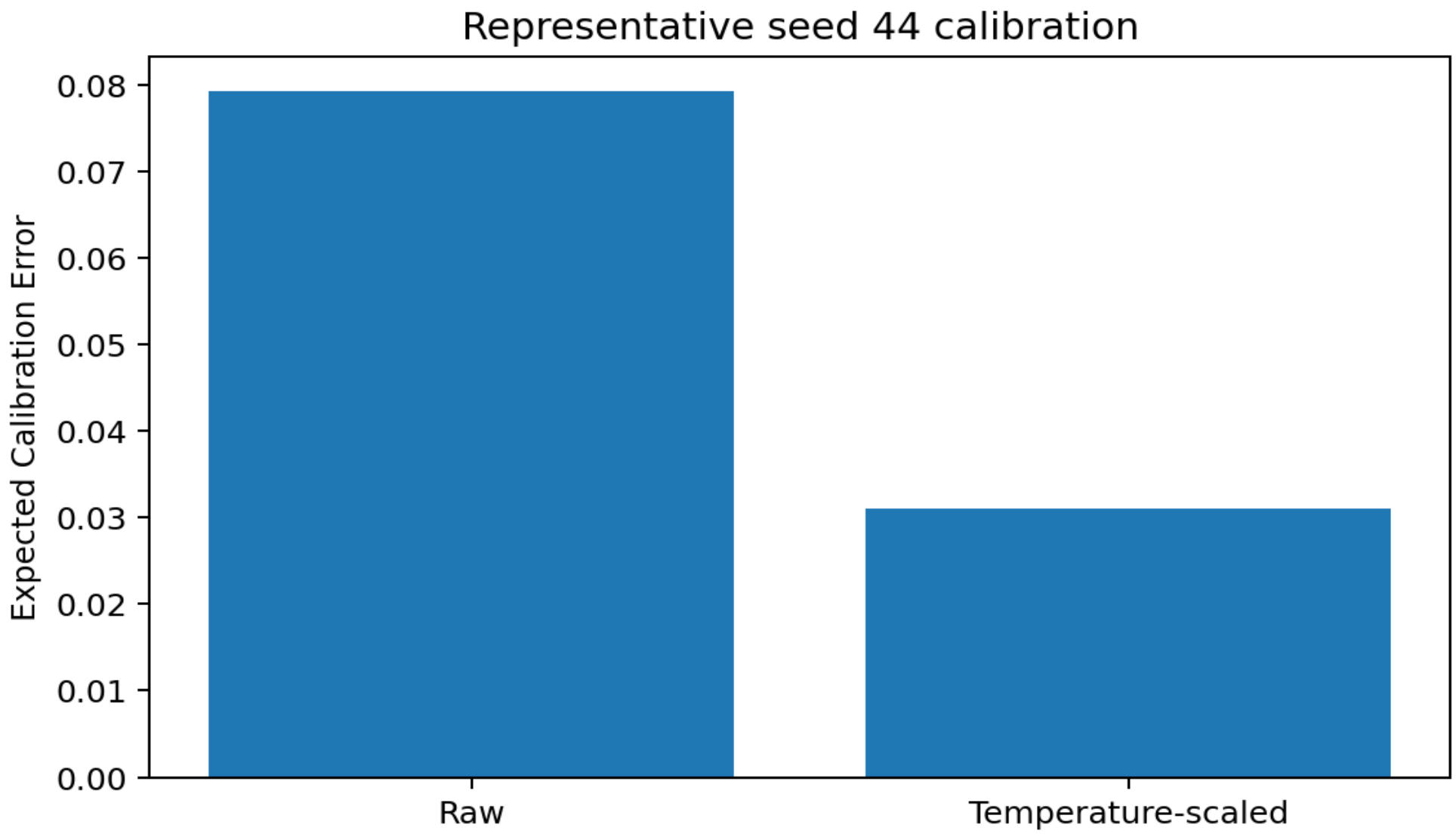

Supplementary Figure S4. Expected Calibration Error before and after validation-fitted temperature scaling for representative seed 44.

S10. Grad-CAM++ quantitative validation

Transformer-compatible Grad-CAM++ was evaluated post hoc on the locked representative model. The analysis included visual explanations, perturbation-based confidence-drop fidelity, transformation stability, and model-randomization sanity checks.

Grad-CAM++ quantity	Result
Mean confidence drop	0.2305
Positive confidence-drop rate	0.8400
Prediction preservation under transformations	0.9750
Mean Pearson correlation	0.9743
Mean Spearman correlation	0.9610
Mean thresholded IoU	0.8612
Mean absolute CAM difference	0.0273

These measurements evaluate model-centered explanation behavior. They do not establish pixel-level lesion localization accuracy because expert lesion masks were not available.

S11. Transformation stability

Transformation	Samples	Prediction preservation rate	Mean Pearson correlation
Brightness	50	1.00	0.9999
Gaussian noise	50	1.00	0.9993
Horizontal flip	50	0.96	0.4463
Translation	50	0.96	0.8696

Photometric perturbations showed very high attribution correlation, whereas geometric transformations produced larger spatial changes. Across the transformation analysis, prediction preservation remained high.

The manuscript's final interpretation therefore treats transformation stability as one component of explanation validation rather than as proof of biological lesion localization.

S11. Grad-CAM++ model-randomization sanity check

Randomized component	Mean Pearson correlation	Mean thresholded IoU	Mean absolute CAM difference
Classification head	0.1071	0.2236	0.2219
Final Swin feature stage	0.3794	0.2970	0.2011
Final two feature stages	0.1317	0.1962	0.2380

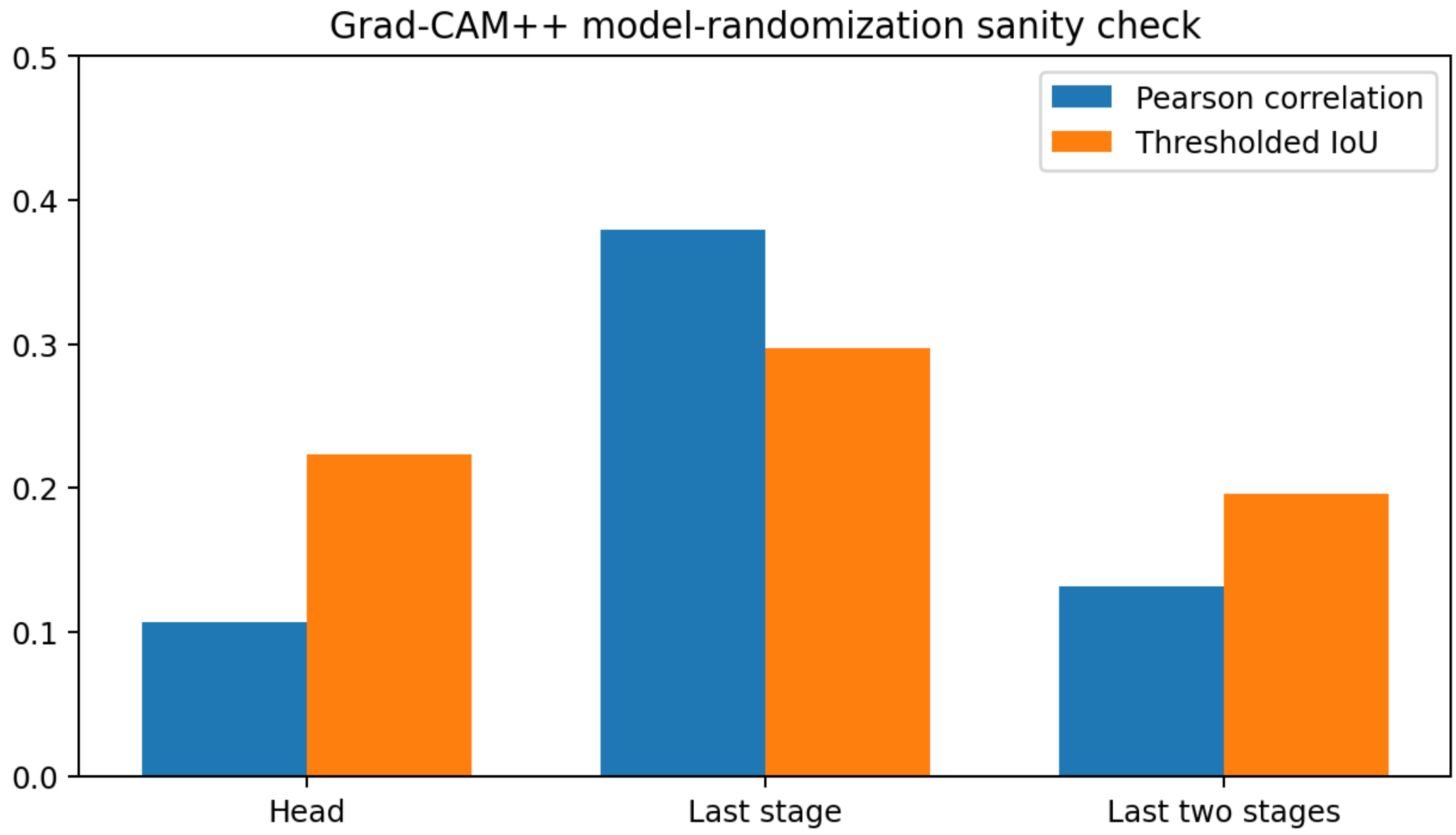

Supplementary Figure S5. Grad-CAM++ model-randomization results relative to the trained model.

The substantial attribution changes after parameter randomization indicate dependence on learned model parameters. The randomization conditions are not interpreted as a monotonic cumulative degradation sequence.

S12. Statistical comparison across the five final models

Cochran's Q was used to compare correctness patterns across the five seeded final models.

Statistic	Result
Cochran's Q	1.2023
p-value	0.877718
Number of final seeded models	5

Pairwise McNemar tests were also computed for all seed pairs, followed by Holm correction for multiple comparisons.

Seed pair	Raw p-value
11 vs 22	0.635828
11 vs 33	0.838820
11 vs 44	1.000000
11 vs 55	0.845019
22 vs 33	0.855536
22 vs 44	0.627103
22 vs 55	0.451381
33 vs 44	0.867939
33 vs 55	0.607591
44 vs 55	0.871415

S12. Interpretation of seeded-model statistics

The Cochran's Q p-value of 0.877718 does not indicate a statistically detectable difference in correctness patterns among the five final seeded models under this test.

The raw pairwise McNemar p-values are all above 0.05; Holm adjustment therefore does not create evidence of pairwise significance. These analyses support reporting the five runs as a stable final model family rather than selecting a seed on held-out test performance.

The ensemble is reported as a probability ensemble of the five already-finalized models; it is not used to reopen development-stage model selection.

S13. Multi-XAI interpretation

The explainability framework uses complementary post-hoc explainers, including Grad-CAM++, SHAP, and LIME, together with deterministic fusion. The explainers are not assumed to be interchangeable or uniformly superior.

Interpretive principle	Supplementary clarification
Complementarity	Different explainers can emphasize different image regions because their mathematical mechanisms differ.
Fusion	Fusion summarizes complementary attribution evidence; it is not automatically more accurate than every indivi
Lesion localization	Highlighted regions are prediction-relevant regions, not expert-validated lesion masks.
Disease severity	Attribution-derived area/burden summaries are not equivalent to agronomic disease severity.

S13. Attribution-burden limitation

The extended analysis explicitly tested whether fused XAI maps could support a quantitative severity-related interpretation. The final decision was **QUALITATIVE_ONLY**.

Decision component	Result
Threshold ranking stable	True
Burden-measure consensus	True
Descriptive health pattern	False
Otsu/MAD alignment with consensus	False
Final decision	QUALITATIVE_ONLY

Recommended interpretation: Multi-XAI fusion is retained for qualitative explanation and relative attribution-burden analysis, but quantitative affected-region percentages should not be emphasized.

This limitation is important because no expert lesion masks or expert severity grades were available. Accordingly, the supplementary evidence does not validate agronomic disease severity.

S14. Reproducibility checklist

Reproducibility item	Recorded setting/evidence
Dataset size	2,815 images; 14 joint species-health classes
Fixed partitions	1,970 / 422 / 423 train/validation/test
Selected preprocessing	Original RGB
Selected architecture	Swin Transformer-Tiny
Input resolution	384 x 384
Enhancement	E0 baseline
Test-time augmentation	Not selected
Final seeds	11, 22, 33, 44, 55
Selection metric	Validation macro-F1
Calibration fit split	Validation only
Representative XAI seed	44
Held-out test used for selection	No
Final pipeline verdict	PASS

The complete executed notebook archive contains the underlying code cells, generated manifests, intermediate reports, and outputs. This supplementary document intentionally presents the publication-relevant evidence in condensed form.

Supplementary file caption and production note

Suggested caption

Supplementary Material 1. Additional experimental details, dataset integrity and leakage-control analyses, validation-only preprocessing and architecture screening, five-seed reproducibility results, locked held-out evaluation, calibration analysis, quantitative explainability validation, statistical analyses, and supporting reproducibility information for the AgroXAI framework.

Suggested response to Frontiers Q25

Response to Q25: Thank you. Supplementary Material 1 has been provided and contains additional experimental, reproducibility, calibration, statistical, and explainability analyses supporting the manuscript. The supplementary file has not been previously deposited elsewhere.

Suggested in-text citation

Additional details on dataset integrity, experimental configuration, reproducibility, calibration, statistical analysis, and explainability validation are provided in **Supplementary Material 1**.

Scope statement

The supplementary material should be interpreted together with the main article. Where a supporting analysis is model-centered, the wording intentionally avoids converting attribution evidence into claims of expert-validated lesion localization or quantitative disease severity.

End of Supplementary Material.
